# Songs tell a story: The Arc of narrative for music

**DOI:** 10.1371/journal.pone.0303188

**Published:** 2024-05-16

**Authors:** Max Alberhasky, Patrick K. Durkee

**Affiliations:** 1 Department of Marketing, California State University Long Beach, Long Beach, CA, United States of America; 2 Department of Psychology, California State University Fresno, Fresno, CA, United States of America; Educational Testing Service (ETS), UNITED STATES

## Abstract

Research suggests that a core lexical structure characterized by words that define plot staging, plot progression, and cognitive tension underlies written narratives. Here, we investigate the extent to which song lyrics follow this underlying narrative structure. Using a text analytic approach and two publicly available datasets of song lyrics including a larger dataset (N = 12,280) and a smaller dataset of greatest hits (N = 2,823), we find that music lyrics tend to exhibit a core Arc of Narrative structure: setting the stage at the beginning, progressing the plot steadily until the end of the song, and peaking in cognitive tension in the middle. We also observe differences in narrative structure based on musical genre, suggesting different genres set the scene in greater detail (Country, Rap) or progress the plot faster and have a higher rate of internal conflict (Pop). These findings add to the evidence that storytelling exhibits predictable language patterns and that storytelling is evident in music lyrics.

## Introduction

Songs both shape and are shaped by our psychology. Music is a human universal with structure and functions that are strikingly similar across cultures, suggesting deep evolutionary roots [1]. Music has the ability to shape our imaginations, emotions, and our identities [2–8]. People spend a substantial amount of their time listening to music. In the United States, the average person over the age of 13 listens to 32 hours of music per week, or 4.5 hours each day which has been steadily increasing over time [9].

Part of the appeal of music may come from the *story*, or narrative, which music conveys. Narrative is a vessel through which musicians can express stories from their lives to their listeners, and these narratives tend to be personal, reveal changes in social attitudes, and be genre-dependent. Musicians can also use storytelling through their songs to express feelings or frustrations in order to make sense of break-ups, or share personal experiences from their own lives which listeners may relate to or learn from. In terms of understanding social attitudes and the collective psyche, recent work finds that there is a gender bias in music in which women are less associated with desirable traits (i.e., competence), although this has decreased in more recent decades and is genre-dependent [10]. Certain genres may tell more or less detailed stories than others, which can be revealed through the song or genre’s lexical structure, drawing on prior literature which suggests that one’s favorite music genre tends to be associated with the person’s cognitive style [11].

Quora.com, a popular online community for asking and answering questions, posed the question, “Do you believe country music tells a story in their songs more than other genres of tunes?”. The top answer to the question is, “In my personal opinion and almost a lifetime of observations (50 years) it does. I profess to like multiple genres of music (especially and mostly country), but country and the blues seem to be best a telling a story and involving connecting to the listener” [12]. Anecdotally and empirically, it stands to reason that different genres of music may, on average, contain different narratives and hence produce different narrative structures.

If musicians express stories about their lives through music and specific genres tend to have collective life stories experienced by the artists, there may be differences in the narratives told between major genres of music [13–17]. Music theorists have had conferences, held discussions/debates, and written books questioning whether one can speak of narrativity in music [18]. Acknowledging that narratives typically elaborate more upon a broader or lengthier story, whereas songs generally manifest in a 3 to 5-minute “mini-narrative” about a particular subject, relationship, or scenario [19], we sought to uncover patterns in story embedded within the narrative of music lyrics. Music can simultaneously enhance storytelling [20,21], such as when the background music of a movie contains a sad song to accompany a character’s misfortune or a feel-good song at the end of the movie as two lovers experience their happily-ever-after moment. In the present research, we examine whether the stories conveyed by song lyrics follow the narrative structure of more extended forms of storytelling (e.g., books, film scripts).

Research in social science studying music and how it relates to the psychology of the individual or the psychology of the group has examined how music can help express our personalities and identities to others [7,8,22,23], evoke specific emotions and feelings [24–30], and aid work ethic or learning and memory [31,32]–though the evidence for this is somewhat unclear [33]. Media such as books, films, or music can serve as a self-reinforcing medium of emotional congruency whereby people prefer listening to sad music while feeling sad [34]. Rather than studying the beat, rhythm, timbre, or other sound-based features of music, we focus specifically on the words extracted from the transcribed lyrics. To the best of our knowledge, we are among the first to examine the Arc of Narrative expressed through the lyrics of music. We aim to contribute to the literature by investigating the lexical structure conveyed through the lyrics of songs.

We help contribute to the psychological understanding and function of music by investigating the features expressed in narrative by music through text analysis of two corpora of lyrics. Building on research which suggests written stories have a core narrative structure, or Arc of Narrative [35], we sought to investigate the extent to which music can be understood as a narrative by utilizing text analysis [36,37]. We investigate narrative structure by examining how three unique yet fundamental aspects of narrative are expressed in song: staging, plot progression, and cognitive tension.

Staging, plot progression, and cognitive tension are extractable via text analysis within the LIWC Arc of Narrative dictionary [35]. The staging variable captures words in which the storyteller provides background information and necessary context. Staging is represented by the frequency of articles, which are used to mark nouns, possessives, and prepositions, which are used to relate nouns to subjects that occur. Examples of words captured by the staging variable include: about, after, and since. This is when the artist may establish names, places, or relationships to provide the listener with contextual information to understand the context of the song or story being told [35].

The plot progression variable essentially captures how the story is moving forward, explaining what familiar people are doing and how they are doing it. Plot progression captures the frequency of words which move the story along from scene-to-scene, captured through function words and pronouns, or auxiliary verbs [35]. Plot progression contains pronouns, negations, conjunctions, auxiliary verbs, and adverbs. Examples of words captured by the plot progression variable include: became, should, and suddenly. Cognitive tension is akin to the frequency of problem-solving, or working through things, which occurs as people try to understand and make sense of events throughout the song.

The cognitive tension variable captures words directly related to internal conflicts experienced by characters, decision-making, and problem-solving. Cognitive tension is adapted from the cognitive processing variable from LIWC (“cogproc”). Examples of words captured by the cognitive tension variable include: alternatives, believe, and deciding. Following prior literature [35], we segmented the lyrics of each song into five equally sized parts to examine how the lexical structure of the lyrics changes throughout the song. The total number of words categorized for the three dictionaries varies between staging (75 words), plot progression (448 words) and cognitive tension (393 words) [35,37].

In the current research, we provide the first direct test of the Arc of Narrative in song lyrics. Additionally, we examine whether there are differences in the narrative structure across musical genres and between segments (i.e., the beginning, middle, and end of the song).

## Materials and methods

We analyzed two publically available datasets of music lyrics. The first dataset [38] contains lyrics from a total of 15,103 songs across six music genres (EDM, Pop, Rap, R&B, Latin and Rock); this dataset includes songs released between the years 1957 and 2020. The second dataset [39] contains lyrics from 3,926 top songs from popular artists across four music genres.

We excluded songs containing lyrics that are not in English and with less than 200 total words, leaving 12,280 songs for analysis in the larger dataset (please see Table 1 in the S1 File for descriptive statistics). After the same exclusions listed for the larger dataset, the smaller dataset contains a total of 2,823 songs with between 500–1,000 songs for each of the four genres listed which are Rock, Country, Rap, and Pop (please see Table 2 in the S1 File for descriptive statistics).

**Fig 1:**
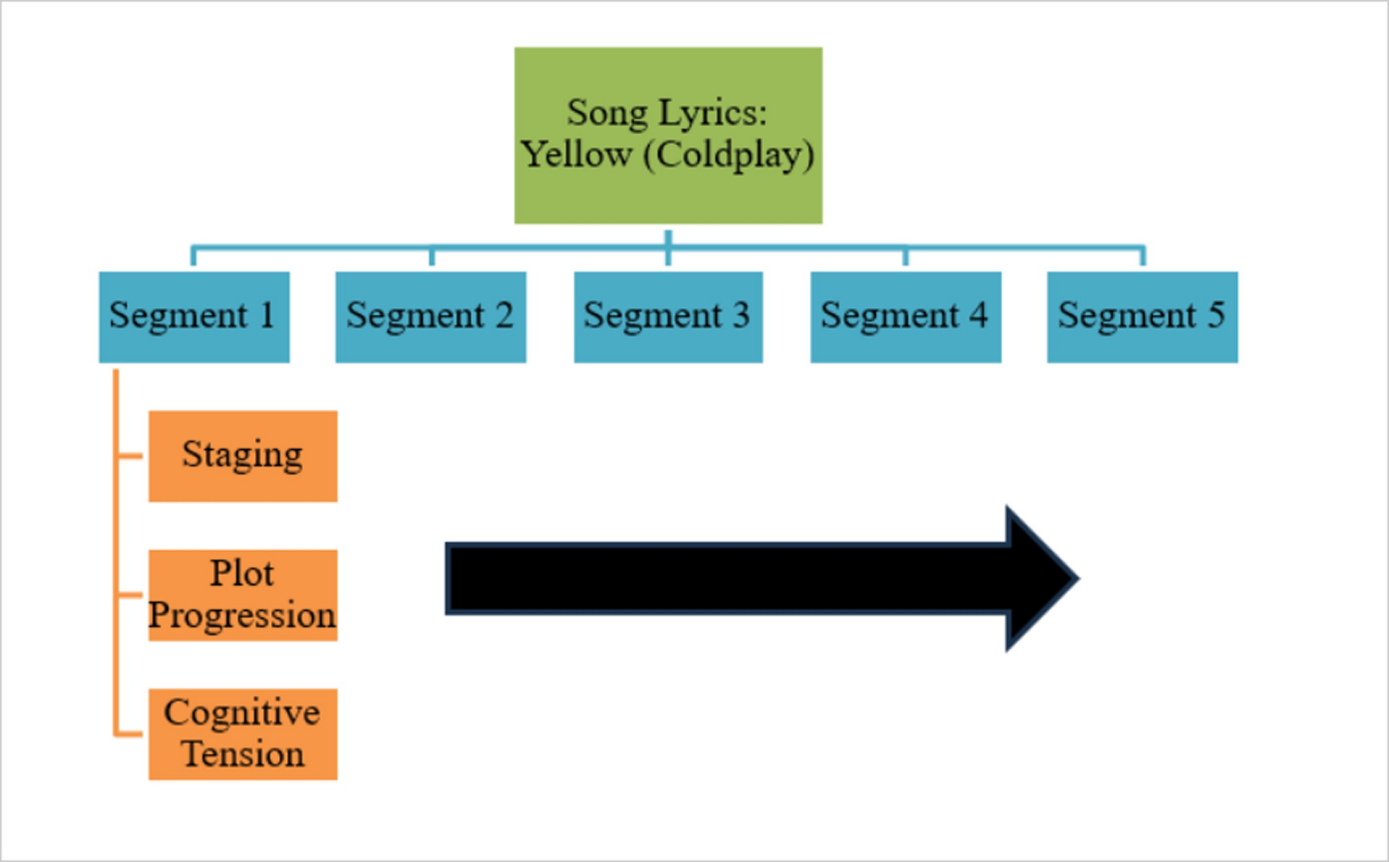
Segmentation strategy and Arc of narrative example.

**Fig 2:**
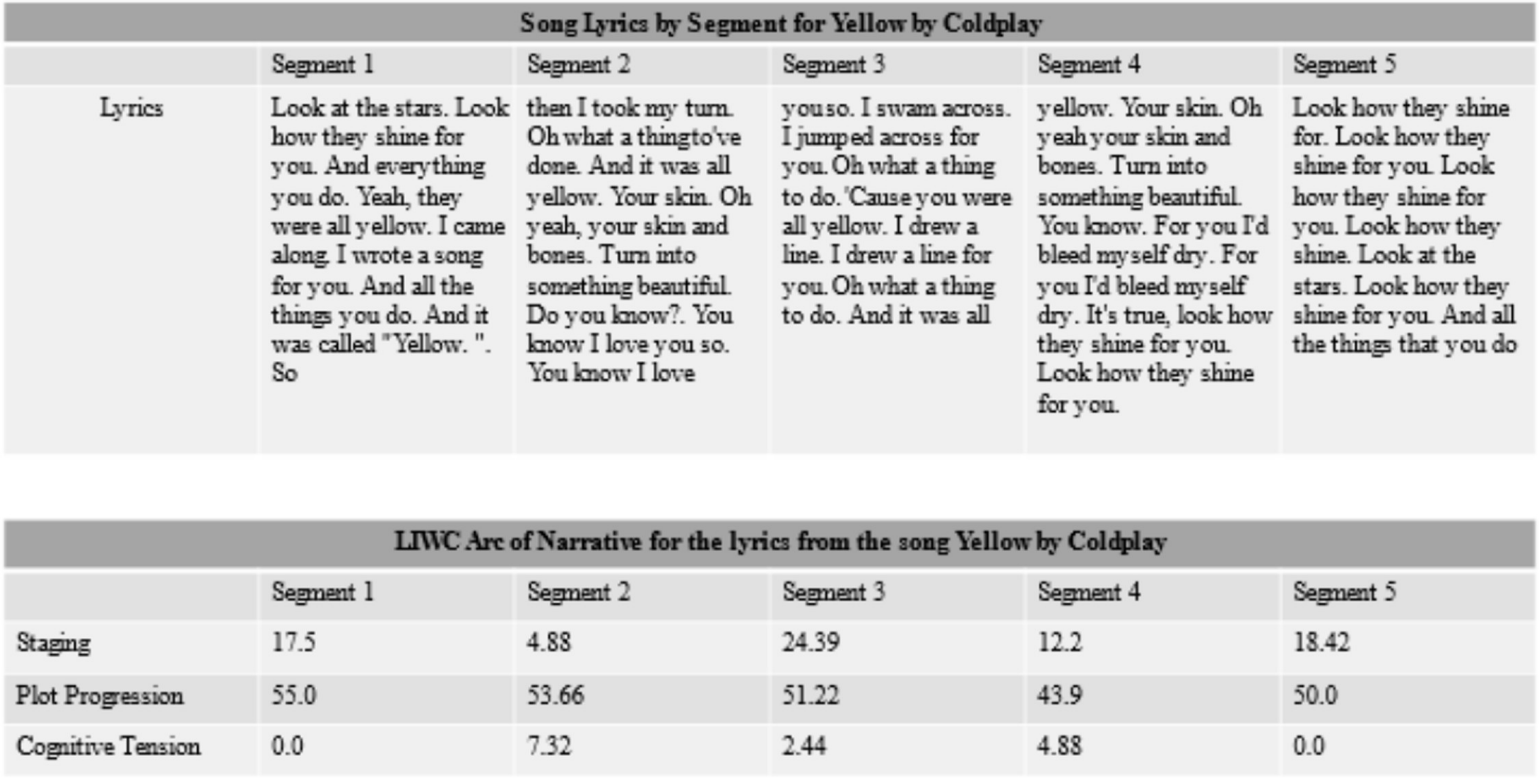
Segmentation and analysis of lyrics example for yellow by coldplay.

**Fig 3:**
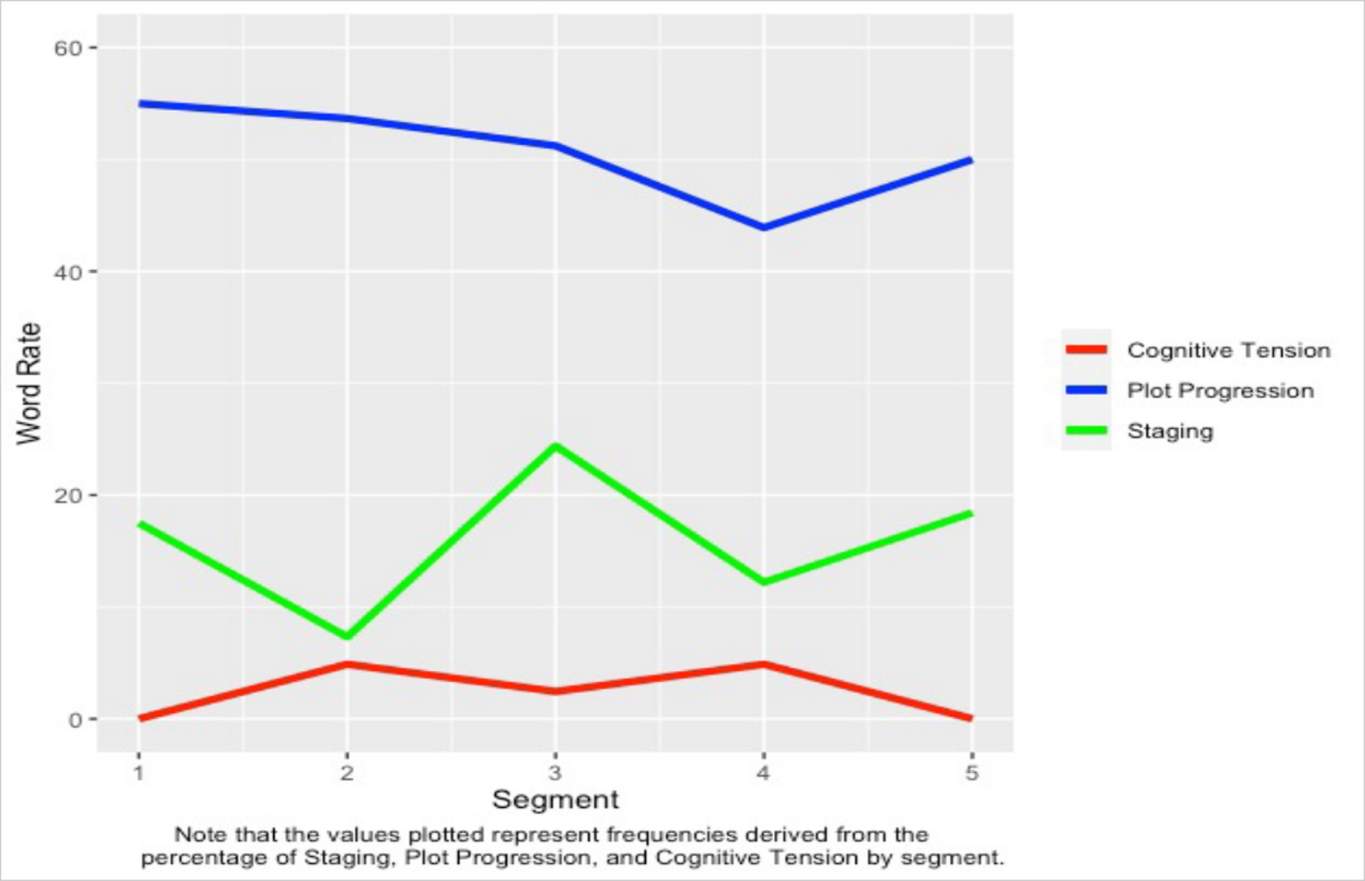
The Arc of narrative plot for yellow by coldplay.

We segmented each song into five equal-sized parts in order to investigate changes in narrative structure as the song progresses. The criterion for segmentation is essentially the number of words contained in the lyrics divided by five. For example, if a song contains 500 words in the lyrics, this song would be segmented into five segments of 100 words each. The segmentation process was done using text analytics software which allows for a given corpus to be segmented into equally sized segments. After segmenting the dataset, we used LIWC to analyze the song lyrics by segment using the Arc of Narrative dictionary to extract the level of staging, plot progression, and cognitive tension. In other words, there are 15 narrative measurements per song (3 variables x 5 segments) that served as our unit of analysis. For an example of the data segmenting and analytical approach we use, please see Figs 1–3 below.

**Fig 4:**
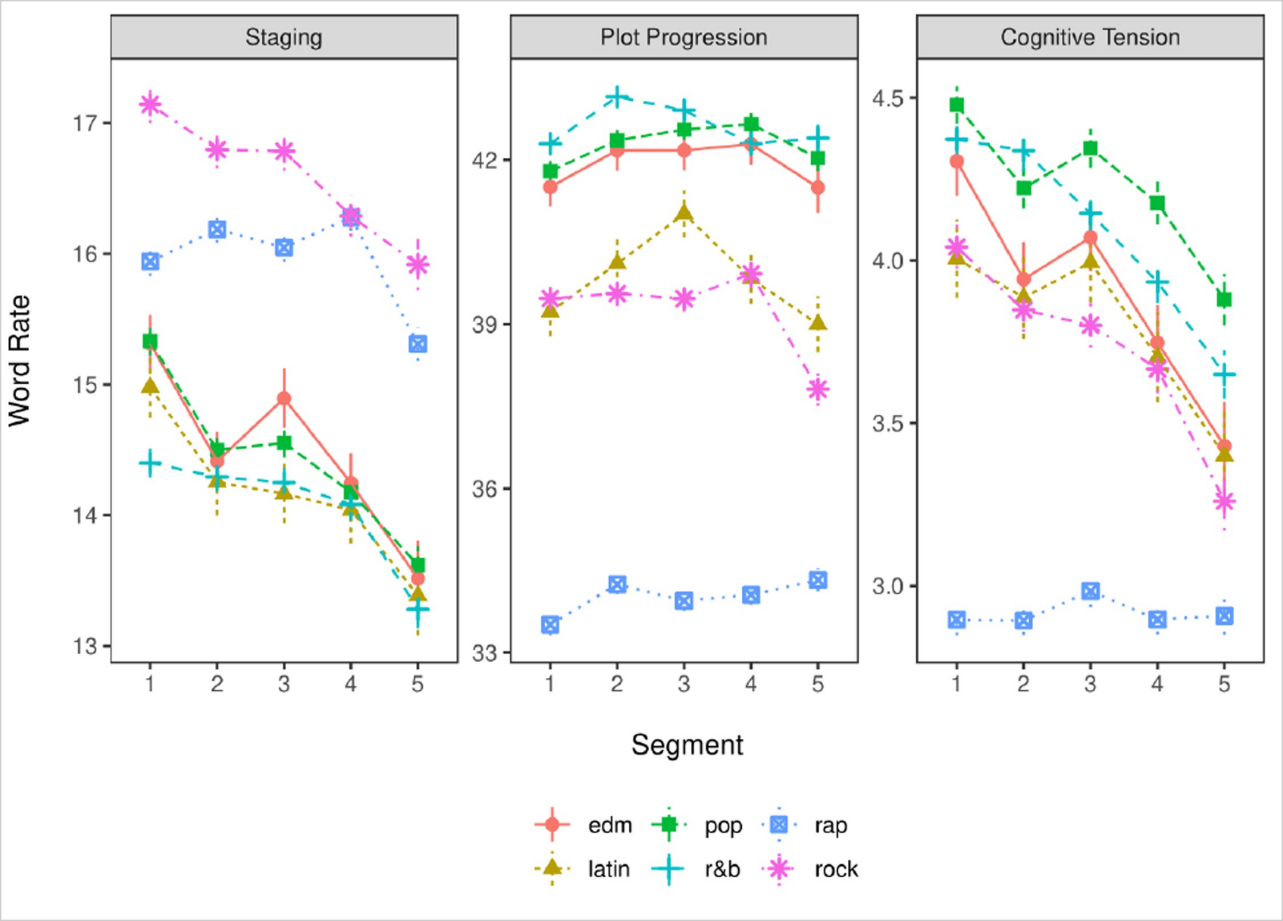
The Arc of narrative for the larger dataset.

We analyzed each song’s lexical structure using the custom LIWC dictionary [35], which is provided on the Open Science Framework (https://osf.io/q2a7m/). The dictionary categories define words associated with staging (i.e., articles and prepositions), plot progression (i.e., pronouns, auxiliary verbs, negations, conjunctions, and non-referential adverbs), and cognitive tension (i.e., words from the standard cognitive processing LIWC dictionary directly related to conflict and problem-solving). Table 1 in S1 File shows descriptive statistics across songs for each genre (see S1 File). Fig 4 shows the Arc of Narrative for the larger dataset.

**Fig 5:**
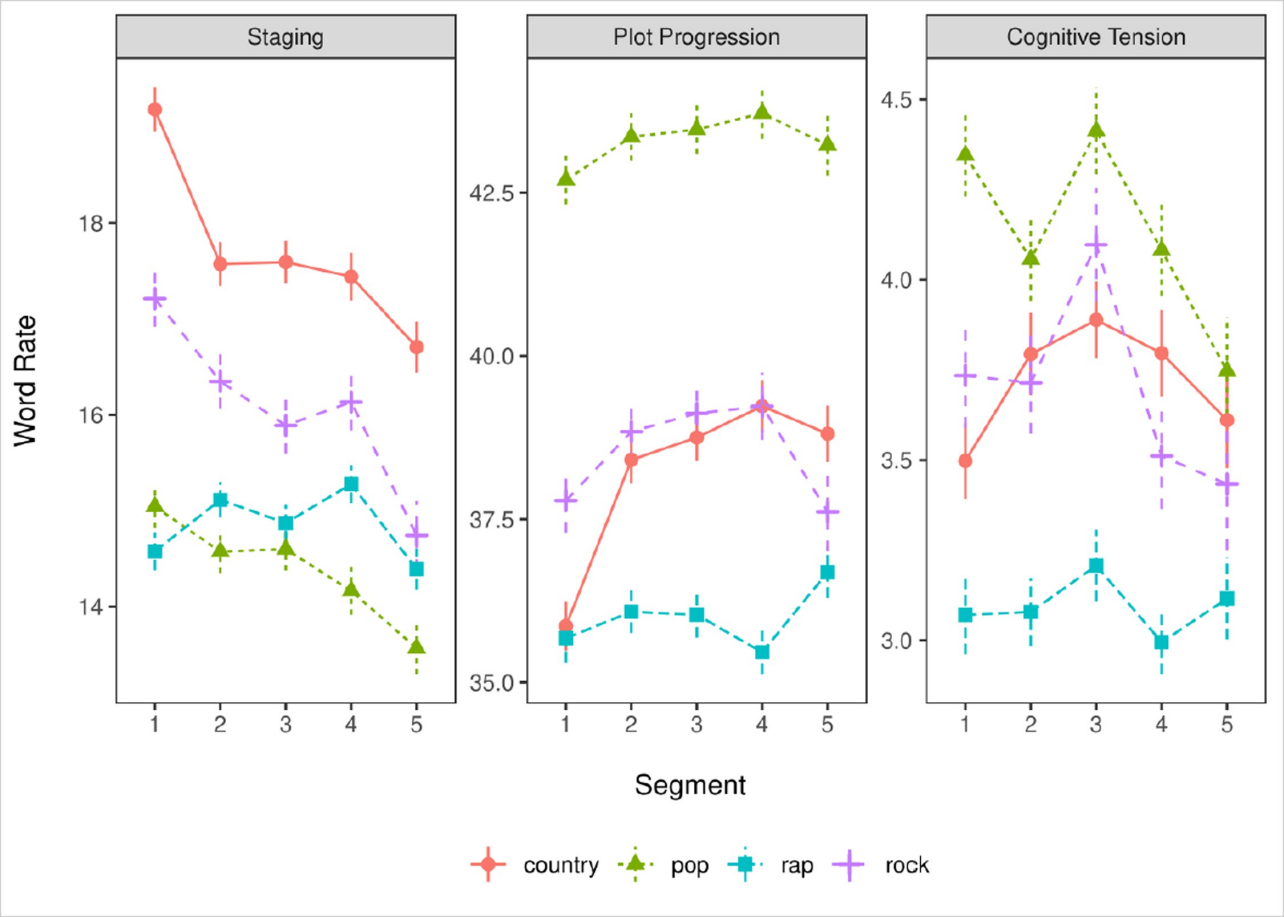
The Arc of narrative for the smaller dataset (top hits).

All analytical methods are identical across both datasets: songs were segmented, run through LIWC to obtain the word counts for each Arc of Narrative category, and analyzed using multilevel models predicting the frequency of words belonging to each category from segment number. The code for the analysis is available on the Open Science Framework (https://osf.io/exa3v/). A summary of results from these analyses are available in Fig 5. Complete pairwise comparisons and descriptive statistics are available in the S1 File.

## Results

Part one of the analysis, using the larger dataset, descriptively looks at the core narrative structure which music exhibits. Part two of the analysis investigates the Arc of Narrative in greater detail via categorizing an Arc of Narrative pattern by genre for a smaller dataset of top hits. In part one of the analysis, we find that staging (scene-setting, which is captured by the presence of articles and prepositions) tends to peak at the beginning and steadily decrease towards the end of the song. We find that plot progression (describing what familiar people are doing and how they are doing it, which is captured by the presence of pronouns, conjunctions, and adverbs) remains fairly steady throughout the first four segments of the song, then decreases in the final segment. Cognitive tension (describing psychological and mental processes) tends to decrease steadily throughout the song, starting with a relatively high amount of tension which gets resolved towards the end of the song. Please see Fig 4 for a depiction that outlines the Arc of Narrative for this larger dataset.

Worth noting is the overall rate, shown on the y-axis, in the figures presented. Plot progression is the most frequently captured variable in both datasets, which is consistent with prior findings of core narrative structure occurring at approximately 40% frequency [35]. Staging occurs significantly less frequently, between 13–17% of word frequencies depending on genre and segment. Finally, cognitive tension is the least frequent element of narrative, occurring at a frequency of 3–5% depending upon genre and segment.

There are several general trends observed from this larger dataset which reveal how the Arc of Narrative unfolds through music lyrics. Across all six genres in this dataset, we find that staging decreases substantially from the beginning of the song to the end of the song. Although this difference is approximately 1–2%, it suggests that the frequency of articles and prepositions decreases as songs progress. The trends revealed through plot progression are a bit less straightforward. The general shape shown in Fig 4 across genres hints that plot progression tends to start low, peak around the middle (somewhere between segment 2–4) and decrease in the final segment as the song concludes. Cognitive tension starts high and decreases steadily and somewhat rapidly from the beginning of the song to the end. Next, we sought to address differences which were produced by genre for this larger dataset.

Of the genres observed in the first dataset, rap music appears to demonstrate the most unique Arc of Narrative. Rap has a relatively high amount of staging compared to other genres (except Rock). However, Rap has substantially lower levels of plot progression and cognitive tension compared to EDM, Pop, Latin, Rock, and R&B. An interpretation for this pattern of results is that Rap music provides more scene setting (i.e., staging) to paint a more vivid picture of the context yet contains relatively less transitions from one scene to another (plot progression) and internal conflict or cognitive processing (cognitive tension) compared to the other music genres in this dataset. Another noteworthy genre-based trend is Rock music, which elicits the highest amount of staging. Rock music then has a large dip in plot progression and cognitive tension during the final segment of songs, which suggests that Rock music often contains issues or problems which become resolved by the end of the song. Indeed, the lexical pattern of plot progression and cognitive tension in general decrease in the final segment of the song–perhaps suggesting conflict resolution as the song concludes.

To examine overall statistical differences in the Arc of Narrative in this larger dataset, we used a multilevel model to predict staging, plot progression, and cognitive tension using segment, genre, and their interaction term. The results show that there is a significant main effect of the segment on the word rate of staging (*F*(4, 48928) = 111.30, *p* < .001), a significant main effect of the genre of music on staging (*F*(5, 12832) = 90.71, *p* < .001). We also observe a significant interaction between segment and genre (*F*(20, 48928) = 5.80, *p* < .001). For plot progression, we also find a significant main effect of segment (*F*(4, 48928) = 27.83, *p* < .001) and a main effect of genre (*F*(5, 12832) = 297.23, *p* < .001). There is a significant interaction between staging and genre on plot progression (*F*(20, 48928) = 6.97, *p* < .001). Finally, we find a similar pattern of results examining cognitive tension with a main effect of segment (*F*(4, 48928) = 82.43, *p* < .001) and of genre (*F*(5, 12832) = 81.18, *p* < .001). The segment of song and music genre also interact with one another significantly for the cognitive tension variable (*F*(20, 48928) = 5.85, *p* < .001). In other words, the Arc of Narrative varies substantially based on which segment is being examined and which genre of music the song belongs to. The interaction between segment and genre suggests that different genres of music may tell different stories at different points throughout the song. All pairwise comparisons at the segment and genre level are available in the S1 File.

In part two of our analysis examining the smaller dataset, results reveal differences between genres. Country music, a genre which is not included in the larger dataset, exhibits the highest amount of staging, which suggests more scene-setting and supports the notion that Country music involves relatively specific or detailed stories compared to other genres as mentioned anecdotally earlier. Pop music has the largest amount of plot progression, suggesting that Pop songs evolve through the story most quickly from start to finish. Finally, we observe that Pop music contains the highest amount of cognitive tension (see Fig 5). This can be interpreted as a storyline that involves making sense of and resolving conflict in situations, events, or relationships.

One difference worth mentioning between datasets is the Arc of Narrative observed for Rap. In the first dataset, Rap has a relatively flat shape between segments for plot progression and cognitive tension (see Fig 4). However, in this smaller dataset, Rap demonstrates a comparatively different shape such that in the final segment plot progression and cognitive tension increase (see Fig 5). To explain this potential discrepancy, we note two important points. The first point is that the first dataset contains substantially more data in terms of songs than the second. Therefore, the Arc of Narrative for Rap in Fig 4 may be more accurate due to the relatively larger sample size. The second point is that the smaller dataset, shown in Fig 5, contains lyrics from top hits. Prior literature suggests that hit songs contain unique or atypical features [40]. Rap songs which are popular appear to conclude the song at a faster pace (moving the plot along quickly in the final segment) and with more cognitive tension (finishing with a problem or dilemma that is unresolved).

We conducted an identical analysis of this smaller dataset using segment, genre, and their interaction term to predict each of the three Arc of Narrative variables in a multilevel model. For staging there is a significant main effect of segment (*F*(4, 11276) = 40.87, *p* < .001), a significant main effect of genre (*F*(3, 2819) = 74.50, *p* < .001), and a significant interaction between factors (*F*(12, 11276) = 6.76, *p* < .001). A similar pattern of results is found for plot progression with the segment affecting the rate at which the plot progresses (*F*(4, 11276) = 17.12, *p* < .001). Genre had a significant impact on plot progression (*F*(3, 2819) = 96.09, *p* < .001), and the interaction between terms is significant (*F*(12, 11276) = 6.49, *p* < .001). Similarly, cognitive tension is predicted by segment (*F*(4, 11276) = 10.63, *p* < .001), genre (*F*(3, 2819) = 24.27, *p* < .001) and the interaction term between factors (*F*(12, 11276) = 2.78, *p* < .001). Because these results converge with the results found for the identical analysis on the larger dataset, the evidence suggests that the narrative of music and how stories are told is shaped substantially based on where the lyrics are positioned (beginning, middle, or end) and the genre the song belongs to. Interestingly, the interaction term is significant which suggests that different genres of music may unfold differently as the song progresses.

Worth noting is the relative strength of the *F* statistics for segment, genre, and their interaction. In both datasets, segment and genre have a substantially stronger impact on staging, plot progression, and cognitive tension compared to their interaction term. This suggests that where the lyrics are located in terms of the overall narrative of the song (i.e, beginning, middle, or end) and which genre the song belongs to (e.g., Rock, Pop, Rap) impact the Arc of Narrative more than their interaction. In other words, even though the interaction between segment and genre is statistically significant in our dataset, this interaction effect is rather small compared to the influence that segment and genre have independently on how narrative is expressed. Please note that all specific comparisons at the individual segment and genre level are available in the S1 File.

## Discussion

It appears that music demonstrates a systematic Arc of Narrative. Song lyrics typically give context and set the stage initially to introduce people, places, and relationships. Song lyrics progress the plot and build up cognitive tension. Cognitive tension peaks in the middle and decreases towards the end, whereas plot progression tends to peak in the fourth stage and drop at the very end. The results suggest that the Arc of Narrative, on aggregate, is genre-dependent, which indicates that songs tell different narratives depending upon their genre. By examining the Arc of Narrative expressed through the lexical structure of song lyrics, our findings contribute to a growing body of empirical research using natural language processing to discover narrative structure [35].

The present research contains practical applications for the music industry and theoretical implications for scholars. Artists and music producers can potentially use the Arc of Narrative to understand how a new song or album compares to their prior work, or the genre more broadly. For example, a country artist who is creating a new album or specific song could compare the narrative structure of their lyrics to the genre as a whole.

Behavioral scientists studying music from a cultural lens could employ natural language processing and Arc of Narrative tools to compare how the lexical structure of songs may be atypical [40] or counterintuitive [41], thus building upon the blueprint we provide in our analysis of the Arc of Narrative for music. Scholars investigating music from a historic [10,42] or cross-cultural perspective [13,43] may also take interest and build upon our findings to understand how the lexical structure of songs has varied over time or geographic region.

An interesting follow-up question for future research would be to investigate whether or not the Arc of Narrative is predictive of a given song’s popularity. Initial research on the Arc of Narrative did not find evidence that story popularity was associated with diversion from the typical narrative structure using content such as books and TED talk transcripts [35]. On the other hand, researchers examining what makes songs catch on finds that popular songs tend to contain more atypicality within their lyrics [40]. For instance, it could be the case that songs with higher staging or cognitive tension tend to be more popular with music genre as a moderating factor. Additional data on song popularity could help to determine whether narrative structure atypicality is associated with popularity and remains a fruitful avenue for future research to pursue.

### Limitations

Several limitations of the present work exist. The first limitation is the question of whether artists are responsible for writing their own lyrics. Artists may produce and perform music containing lyrics that they did not personally write themselves. Although many songs are not written by the artists themselves, they may still permeate the music industry and contribute to the genre. An interesting avenue for future research could be to examine how narrative structure differs depending on whether the artist organically produced the lyrics themselves (versus having the lyrics produced by someone else or an Artificial Intelligence software).

The second limitation of this work comes from the connection between using five segments to divide each song into. The rationale for this methodological choice draws from prior literature on the lexical structure of narrative [35]. However, one could make the argument that song lyrics are more succinct compared to larger corpora (e.g., books, movie scripts) and may lead to choosing a different methodological approach. For instance, our segmentation strategy of five equally sized segments based on word count could be replaced with a segmentation strategy more mindful of verse, chorus, repetitiveness, or other relevant factors. While we chose to follow prior literature for our analyses, a potential limitation and question for future research could be to examine how narrative structure may change using a different segmentation strategy.

The third limitation of the present work comes from the data itself. Music lyrics are more redundant than other narrative vessels, and lyrics may contain more slang, different vocabulary, and varying levels of grammatical correctness. It is possible that different genres of music contain different norms or expectations, implicitly or explicitly, for how the lyrics should unfold. Although this fits our findings or argument to a certain extent, genre norms could also contribute to our findings. Inherent to the way songs tend to be written, the words in a song are likely more repetitive than those of articles, books, scripts, or other texts. We chose to include repetitive stanzas/phrases in our datasets to maintain the integrity of the original song. Artists may repeat key stanzas to emphasize points or features of their message as part of how they wish to convey their story. However, one could argue that removing repetitive lyrics is valid. Future research could consider removing such redundancies to investigate the Arc of Narrative for song lyrics to compare how the lexical structure unfolds with our findings.

## Conclusions

Music and storytelling are two fundamental facets of human psychology and culture. Using a text analytic approach, we find songs tend to exhibit an underlying core narrative structure similar to other storytelling mediums. Moreover, different genres of music systematically differ in the extent to which different elements of the core narrative structure are emphasized. Other approaches like interviews/self-report, EEG, FMRI, and machine learning have made important strides in evaluating music features and preferences among listeners [4–7,26,43,44]. Using natural language processing, we add to the conversation by creating an outline for the Arc of Narrative expressed through music lyrics. We encourage future research to continue the investigation of the stories that songs express to listeners through their lyrics.

## Supporting information

S1 File (DOCX)

## References

[1] Mehr SA, Singh M, Knox D, Ketter DM, Pickens-Jones D, Atwood S, et al. Universality and diversity in human song. Science. 2019;366: eaax0868. doi: 10.1126/science.aax0868 31753969 PMC7001657

[2] Margulis EH, McAuley JD. Using music to probe how perception shapes imagination. Trends in Cognitive Sciences. 2022 Oct 1;26(10):829–831. doi: 10.1016/j.tics.2022.07.011 35965164

[3] Juslin PN, Liljeström S, Västfjäll D, Barradas G, Silva A. An experience sampling study of emotional reactions to music: Listener, music, and situation. Emotion. 2008;8: 668–683. doi: 10.1037/a0013505 18837617

[4] Koelsch S. Brain correlates of music-evoked emotions. Nature Reviews Neuroscience. 2014;15: 170–180. doi: 10.1038/nrn3666 24552785

[5] Anderson I, Gil S, Gibson C, Wolf S, Shapiro W, Semerci O, et al. Just the way you are: Linking music listening on Spotify and personality. Social Psychological and Personality Science. 2021 May;12(4):561–72. 10.1177/1948550620923228.

[6] Rentfrow PJ. The Role of Music in Everyday Life: Current Directions in the Social Psychology of Music. Social and Personality Psychology Compass. 2012;6: 402–416. 10.1111/j.1751-9004.2012.00434.x.

[7] Rentfrow PJ, Gosling SD. The do re mi’s of everyday life: The structure and personality correlates of music preferences. Journal of Personality and Social Psychology. 2003;84: 1236–1256. doi: 10.1037/0022-3514.84.6.1236 12793587

[8] Rentfrow PJ, Gosling SD. Message in a Ballad. Psychological Science. 2006;17: 236–242. 10.1111/j.1467-9280.2006.01691.x. 16507064

[9] Christman E, Christman E. Nielsen 360 Study Finds Consumers Love Streaming Music, But Radio Still Strong. In: Billboard [Internet]. 15 Nov 2017. Available: https://www.billboard.com/pro/nielsen-music-360-2017-report-streaming/.

[10] Boghrati R, Berger J. Quantifying cultural change: Gender bias in music. Journal of Experimental Psychology: General. 2023 Apr 13.2591–2602. doi: 10.1037/xge0001412 37053396

[11] Greenberg DM, Baron-Cohen S, Stillwell DJ, Kosinski M, Rentfrow PJ (2015) Musical Preferences are Linked to Cognitive Styles. PLoS ONE 10(7): e0131151. doi: 10.1371/journal.pone.0131151 26200656 PMC4511638

[12] Nabors Jack. Do you believe country music tells a story in their songs more than other genres of tune? [Internet]. Quora.com; 10 Aug 2019. Available: https://www.quora.com/Do-you-believe-country-music-tells-a-story-in-their-songs-more-than-other-genres-of-tunes.

[13] Cowen AS, Fang X, Sauter D, Keltner D. What music makes us feel: At least 13 dimensions organize subjective experiences associated with music across different cultures. Proceedings of the National Academy of Sciences. 2020;117: 1924–1934. doi: 10.1073/pnas.1910704117 31907316 PMC6995018

[14] Fritz T. Universal Recognition of Three Basic Emotions in Music. Current Biology. 2009;19: 573–576. doi: 10.1016/j.cub.2009.02.058 19303300

[15] Harwood DL. Universals in Music: A Perspective from Cognitive Psychology. Ethnomusicology. 1976;20: 521. 10.2307/851047.

[16] McPherson MJ, Dolan SE, Durango A, Ossandon T, Valdés J, Undurraga EA, et al. Perceptual fusion of musical notes by native Amazonians suggests universal representations of musical intervals. Nature Communications. 2020;11. 10.1038/s41467-020-16448-6. PMC7270137 32493923

[17] Savage PE, Brown S, Sakai E, Currie TE. Statistical universals reveal the structures and functions of human music. Proceedings of the National Academy of Sciences. 2015;112: 8987–8992. doi: 10.1073/pnas.1414495112 26124105 PMC4517223

[18] Nattiez J-J. Can One Speak of Narrativity in Music? Journal of the Royal Musical Association. 1990;115: 240–257. 10.1093/jrma/115.2.240.

[19] Nicholls D. Narrative Theory as an Analytical Tool in the Study of Popular Music Texts. Music and Letters. 2007;88: 297–315. 10.1093/ml/gcm006.

[20] Costabile KA, Terman AW. Effects of Film Music on Psychological Transportation and Narrative Persuasion. Basic and Applied Social Psychology. 2013;35: 316–324. 10.1080/01973533.2013.785398.

[21] Hung K. Narrative Music in Congruent and Incongruent TV Advertising. Journal of Advertising. 2000;29: 25–34. 10.1080/00913367.2000.10673601.

[22] Berger J, Heath C. Where Consumers Diverge from Others: Identity Signaling and Product Domains. Journal of Consumer Research. 2007;34: 121–134. 10.1086/519142.

[23] Jacobson J, Hall S, Gay PD. Questions of Cultural Identity. The British Journal of Sociology. 1997;48: 153. 10.2307/591920.

[24] Hunter PG, Schellenberg EG, Schimmack U. Feelings and perceptions of happiness and sadness induced by music: Similarities, differences, and mixed emotions. Psychology of Aesthetics, Creativity, and the Arts. 2010;4: 47–56. 10.1037/a0016873.

[25] Juslin PN, Laukka P. Expression, Perception, and Induction of Musical Emotions: A Review and a Questionnaire Study of Everyday Listening. Journal of New Music Research. 2004;33: 217–238. 10.1080/0929821042000317813.

[26] Lin Yuan-Pin, Wang Chi-Hong, Jung Tzyy-Ping, Wu Tien-Lin, Jeng Shyh-Kang, Duann Jeng-Ren, et al. EEG-Based Emotion Recognition in Music Listening. IEEE Transactions on Biomedical Engineering. 2010;57: 1798–1806. doi: 10.1109/TBME.2010.2048568 20442037

[27] Markowitz DM, Hancock JT. The 27 Club: Music Lyrics Reflect Psychological Distress. Communication Reports. 2016;30: 1–13. 10.1080/08934215.2016.1210663.

[28] Vuoskoski JK, Eerola T. Measuring music-induced emotion. Musicae Scientiae. 2011;15: 159–173. 10.1177/1029864911403367.

[29] Thoma MV, La Marca R, Brönnimann R, Finkel L, Ehlert U, Nater UM. The Effect of Music on the Human Stress Response. PLoS ONE. 2013;8: e70156. doi: 10.1371/journal.pone.0070156 23940541 PMC3734071

[30] Pereira CS, Teixeira J, Figueiredo P, Xavier J, Castro SL, Brattico E. Music and Emotions in the Brain: Familiarity Matters. PLoS ONE. 2011;6: e27241. doi: 10.1371/journal.pone.0027241 22110619 PMC3217963

[31] Frischen U, Schwarzer G, Degé F. Music lessons enhance executive functions in 6- to 7-year-old children. Learning and Instruction. 2021;74: 101442. 10.1016/j.learninstruc.2021.101442.

[32] Lesiuk T. The effect of music listening on work performance. Psychology of Music. 2005;33: 173–191. 10.1177/0305735605050650.

[33] Mehr SA, Schachner A, Katz RC, Spelke ES. Two Randomized Trials Provide No Consistent Evidence for Nonmusical Cognitive Benefits of Brief Preschool Music Enrichment. PLoS ONE. 2013;8: e82007. doi: 10.1371/journal.pone.0082007 24349171 PMC3859544

[34] Van den Tol AJM, Edwards J. Exploring a rationale for choosing to listen to sad music when feeling sad. Psychology of Music. 2011;41: 440–465. 10.1177/0305735611430433.

[35] Boyd RL, Blackburn KG, Pennebaker JW. The narrative arc: Revealing core narrative structures through text analysis. Science Advances. 2020;6: eaba2196. doi: 10.1126/sciadv.aba2196 32821822 PMC7413736

[36] Boyd R. Data Analytics in Digital Humanities. Cham: Springer International Publishing; 2017. 10.1007/978-3-319-54499-1.

[37] Pennebaker J, Boyd R, Jordan K, Blackburn K. The Development and Psychometric Properties of LIWC2015. 2015.

[38] Audio features and lyrics of Spotify songs. In: www.kaggle.com [Internet]. [cited 19 Jun 2023]. Available: https://www.kaggle.com/imuhammad/audio-features-and-lyrics-of-spotify-songs?select=spotify_songs.csv.

[39] Goldman J. JacobGo/nltk-lyric-corpus. In: GitHub [Internet]. 1 Mar 2022 [cited 19 Jun 2023]. Available: https://github.com/JacobGo/nltk-lyric-corpus.

[40] Berger J, Packard G. Are Atypical Things More Popular? Psychological Science. 2018;29: 1178–1184. doi: 10.1177/0956797618759465 29671695

[41] Norenzayan A, Atran S, Faulkner J, Schaller M. Memory and mystery: The cultural selection of minimally counterintuitive narratives. Cognitive Science. 2006 May 6;30(3):531–53. doi: 10.1207/s15516709cog0000_68 21702824

[42] Mauch M, MacCallum RM, Levy M, Leroi AM. The evolution of popular music: USA 1960–2010. Royal Society open science. 2015 May 6;2(5):150081. doi: 10.1098/rsos.150081 26064663 PMC4453253

[43] Greenberg DM, Wride SJ, Snowden DA, Spathis D, Potter J, Rentfrow PJ. Universals and variations in musical preferences: A study of preferential reactions to Western music in 53 countries. Journal of Personality and Social Psychology. 2022 Feb;122(2):286. doi: 10.1037/pspp0000397 35130023

[44] Piazza EA, Sweeny TD, Wessel D, Silver MA, Whitney D. Humans Use Summary Statistics to Perceive Auditory Sequences. Psychological Science. 2013;24: 1389–1397. doi: 10.1177/0956797612473759 23761928 PMC4381997

