# Supplementary material for: Songs tell a story: The Arc of narrative for music
Source: PLoS One. 2024 May 16;19(5):e0303188. doi: 10.1371/journal.pone.0303188 (PMC11098490; doi:10.1371/journal.pone.0303188)
Supplement: S1 File — (DOCX) [file pone.0303188.s001.docx]

Table of Contents

[Descriptive statistics for each dataset 3](#_Toc162611186)

[Larger dataset pairwise comparisons 4](#_Toc162611187)

[Pairwise comparisons of word rates across genres within segment 5](#_Toc162611188)

[Pairwise comparisons of word rates across segments within genre 11](#_Toc162611189)

[Smaller dataset pairwise comparisons 18](#_Toc162611190)

[Pairwise comparisons of word rates across genres within segment 19](#_Toc162611191)

[Pairwise comparisons of word rates across segments within genre 22](#_Toc162611192)

[Table 1. Means and (standard deviations) of narrative components, segment length, and total word count by genre for the larger dataset. 3](#_Toc162610392)

[Table 2. Means and (standard deviations) of narrative components, segment length, and total word count by genre for the smaller dataset. 3](#_Toc162610393)

[Table 3. Pairwise comparisons of Cognitive Tension word rates within segments between genres (larger dataset) 5](#_Toc162610394)

[Table 4. Pairwise comparisons of Plot Progression word rates within segments between genres (larger dataset) 7](#_Toc162610395)

[Table 5. Pairwise comparisons of Staging word rates within segments between genres (larger dataset) 9](#_Toc162610396)

[Table 6. Pairwise comparisons of Cognitive Tension word rates within genres between segments (larger dataset) 11](#_Toc162610397)

[Table 7. Pairwise comparisons of Plot Progression word rates within genres between segments (larger dataset) 13](#_Toc162610398)

[Table 8. Pairwise comparisons of Staging word rates within genres between segments (larger dataset) 15](#_Toc162610399)

[Table 9. Pairwise comparisons of Cognitive Tension word rates within segments between genres (smaller dataset) 19](#_Toc162610400)

[Table 10. Pairwise comparisons of Plot Progression word rates within segments between genres (smaller dataset) 20](#_Toc162610401)

[Table 11. Pairwise comparisons of Staging word rates within segments between genres (smaller dataset) 21](#_Toc162610402)

[Table 12. Pairwise comparisons of Cognitive Tension word rates within genres between segments (smaller dataset) 22](#_Toc162610403)

[Table 13. Pairwise comparisons of Plot Progression word rates within genres between segments (smaller dataset) 24](#_Toc162610404)

[Table 14. Pairwise comparisons of Staging word rates within genres between segments (smaller dataset) 26](#_Toc162610405)

## Descriptive statistics for each dataset

Table 1. Means and (standard deviations) of narrative components, segment length, and total word count by genre for the larger dataset.

| **Genre** | **Corpus Size** | **Segment Length (words)** | **Total Words** | **Staging** | **Plot Progression** | **Cognitive Tension** |
| --- | --- | --- | --- | --- | --- | --- |
| EDM* | 1118 | 72.02 (28.89) | 353.29 (139.32) | 14.48 (8.00) | 41.93 (13.07) | 3.90 (3.90) |
| Latin | 706 | 92.04 (60.98) | 443.27 (278.81) | 14.16 (6.82) | 39.85 (12.31) | 3.80 (3.50) |
| Pop | 3157 | 107.31 (92.45) | 515.71 (427.55) | 14.44 (6.81) | 42.28 (11.45) | 4.22 (3.69) |
| R&B* | 2675 | 89.64 (41.76) | 437.76 (201.82) | 14.06 (6.23) | 42.61 (11) | 4.09 (3.36) |
| Rap | 2339 | 143.78 (88.04) | 676.28 (393.95) | 15.96 (5.39) | 34.02 (9.38) | 2.92 (2.4) |
| Rock | 2285 | 67.62 (30.75) | 332.34 (144.76) | 16.59 (7.56) | 39.25 (12.22) | 3.72 (3.55) |
| Across Genres | 12280 | 98.93 (72.04) | 476.25 (330.45) | 15.03 (6.78) | 40.04 (11.79) | 3.8 (3.42) |

*EDM is electronic dance music, R&B is rhythm and blues. The values for Staging, Plot Progression, and Cognitive Tension are the average frequencies, or word rates, with which each variable occurs in the respective genre.

Table 2. Means and (standard deviations) of narrative components, segment length, and total word count by genre for the smaller dataset.

| **Genre** | **Corpus Size** | **Segment Length (words)** | **Total Words** | **Staging** | **Plot Progression** | **Cognitive Tension** |
| --- | --- | --- | --- | --- | --- | --- |
| Country | 773 | 56.57 (11.85) | 282.86 (59.01) | 17.70 (6.70) | 38.21 (10.85) | 3.72 (3.23) |
| Pop | 763 | 75.60 (35.57) | 377.98 (177.80) | 14.39 (6.62) | 43.29 (10.93) | 4.13 (3.49) |
| Rap | 754 | 105.68 (40.52) | 528.40 (202.54) | 14.85 (5.43) | 35.99 (9.82) | 3.09 (2.78) |
| Rock | 533 | 58.23 (19.62) | 291.13 (97.98) | 16.06 (7.12) | 38.52 (11.94) | 3.70 (3.59) |
| Across Genres | 2823 | 75.14 (35.9) | 375.71 (179.45) | 15.73 (6.59) | 39.05 (11.17) | 3.66 (3.29) |

# Larger dataset pairwise comparisons

*Note:* All p-values are corrected for multiple tests using Tukey’s method.

## Pairwise comparisons of word rates across genres within segment

Table 3. Pairwise comparisons of Cognitive Tension word rates within segments between genres (larger dataset)

| contrast | Segment | estimate | SE | df | t.ratio | p.value |
| --- | --- | --- | --- | --- | --- | --- |
| edm - latin | 1 | 0.352 | 0.162 | 33500.479 | 2.165 | 0.254 |
| edm - pop | 1 | -0.148 | 0.118 | 33302.767 | -1.253 | 0.81 |
| edm - r&b | 1 | -0.05 | 0.121 | 33244.689 | -0.411 | 0.999 |
| edm - rap | 1 | 1.42 | 0.123 | 33680.217 | 11.575 | < .001 |
| edm - rock | 1 | 0.293 | 0.124 | 33087.034 | 2.37 | 0.167 |
| latin - pop | 1 | -0.499 | 0.14 | 33654.298 | -3.555 | 0.005 |
| latin - r&b | 1 | -0.401 | 0.143 | 33600.903 | -2.81 | 0.056 |
| latin - rap | 1 | 1.068 | 0.145 | 33907.686 | 7.385 | < .001 |
| latin - rock | 1 | -0.059 | 0.145 | 33473.571 | -0.405 | 0.999 |
| pop - r&b | 1 | 0.098 | 0.089 | 33411.44 | 1.105 | 0.879 |
| pop - rap | 1 | 1.567 | 0.092 | 34190.101 | 17.123 | < .001 |
| pop - rock | 1 | 0.44 | 0.093 | 33116.499 | 4.751 | < .001 |
| r&b - rap | 1 | 1.469 | 0.095 | 34028.227 | 15.444 | < .001 |
| r&b - rock | 1 | 0.342 | 0.096 | 33039.374 | 3.557 | 0.005 |
| rap - rock | 1 | -1.127 | 0.099 | 33718.796 | -11.396 | < .001 |
| edm - latin | 2 | 0.077 | 0.161 | 32720.778 | 0.481 | 0.997 |
| edm - pop | 2 | -0.279 | 0.117 | 32555.574 | -2.391 | 0.159 |
| edm - r&b | 2 | -0.39 | 0.12 | 32494.748 | -3.261 | 0.014 |
| edm - rap | 2 | 1.043 | 0.122 | 32918.129 | 8.574 | < .001 |
| edm - rock | 2 | 0.101 | 0.123 | 32388.434 | 0.82 | 0.964 |
| latin - pop | 2 | -0.356 | 0.139 | 32940.697 | -2.558 | 0.108 |
| latin - r&b | 2 | -0.467 | 0.142 | 32884.272 | -3.297 | 0.013 |
| latin - rap | 2 | 0.965 | 0.144 | 33181.49 | 6.728 | < .001 |
| latin - rock | 2 | 0.023 | 0.144 | 32792.913 | 0.16 | 1 |
| pop - r&b | 2 | -0.111 | 0.088 | 32852.315 | -1.256 | 0.809 |
| pop - rap | 2 | 1.322 | 0.091 | 33597.842 | 14.532 | < .001 |
| pop - rock | 2 | 0.38 | 0.092 | 32631.859 | 4.118 | 0.001 |
| r&b - rap | 2 | 1.433 | 0.095 | 33420.19 | 15.158 | < .001 |
| r&b - rock | 2 | 0.49 | 0.096 | 32531.313 | 5.124 | < .001 |
| rap - rock | 2 | -0.942 | 0.098 | 33180.126 | -9.585 | < .001 |
| edm - latin | 3 | 0.095 | 0.161 | 32444.04 | 0.589 | 0.992 |
| edm - pop | 3 | -0.268 | 0.116 | 32315.531 | -2.3 | 0.194 |
| edm - r&b | 3 | -0.07 | 0.119 | 32222.946 | -0.59 | 0.992 |
| edm - rap | 3 | 1.088 | 0.121 | 32689.94 | 8.969 | < .001 |
| edm - rock | 3 | 0.274 | 0.122 | 32176.58 | 2.241 | 0.219 |
| latin - pop | 3 | -0.362 | 0.139 | 32673.74 | -2.607 | 0.095 |
| latin - r&b | 3 | -0.165 | 0.141 | 32595.589 | -1.167 | 0.853 |
| latin - rap | 3 | 0.993 | 0.143 | 32924.466 | 6.943 | < .001 |
| latin - rock | 3 | 0.179 | 0.144 | 32548.2 | 1.247 | 0.814 |
| pop - r&b | 3 | 0.197 | 0.088 | 32609.343 | 2.247 | 0.216 |
| pop - rap | 3 | 1.356 | 0.091 | 33431.219 | 14.931 | < .001 |
| pop - rock | 3 | 0.542 | 0.092 | 32492.35 | 5.886 | < .001 |
| r&b - rap | 3 | 1.158 | 0.094 | 33197.205 | 12.286 | < .001 |
| r&b - rock | 3 | 0.344 | 0.095 | 32335.058 | 3.606 | 0.004 |
| rap - rock | 3 | -0.814 | 0.098 | 33046.732 | -8.293 | < .001 |
| edm - latin | 4 | 0.056 | 0.161 | 32659.004 | 0.351 | 0.999 |
| edm - pop | 4 | -0.415 | 0.117 | 32593.961 | -3.558 | 0.005 |
| edm - r&b | 4 | -0.185 | 0.12 | 32500.503 | -1.548 | 0.633 |
| edm - rap | 4 | 0.856 | 0.122 | 32967.093 | 7.037 | < .001 |
| edm - rock | 4 | 0.081 | 0.123 | 32416.329 | 0.662 | 0.986 |
| latin - pop | 4 | -0.472 | 0.139 | 32859.608 | -3.389 | 0.009 |
| latin - r&b | 4 | -0.241 | 0.142 | 32783.841 | -1.706 | 0.528 |
| latin - rap | 4 | 0.8 | 0.143 | 33114.684 | 5.578 | < .001 |
| latin - rock | 4 | 0.025 | 0.144 | 32712.322 | 0.171 | 1 |
| pop - r&b | 4 | 0.23 | 0.088 | 32866.784 | 2.615 | 0.093 |
| pop - rap | 4 | 1.272 | 0.091 | 33689.303 | 13.968 | < .001 |
| pop - rock | 4 | 0.497 | 0.092 | 32684.767 | 5.384 | < .001 |
| r&b - rap | 4 | 1.041 | 0.095 | 33455.347 | 11.014 | < .001 |
| r&b - rock | 4 | 0.266 | 0.096 | 32532.259 | 2.781 | 0.06 |
| rap - rock | 4 | -0.775 | 0.098 | 33247.528 | -7.879 | < .001 |
| edm - latin | 5 | 0.038 | 0.162 | 33364.102 | 0.232 | 1 |
| edm - pop | 5 | -0.422 | 0.118 | 33228.026 | -3.588 | 0.004 |
| edm - r&b | 5 | -0.2 | 0.12 | 33134.877 | -1.66 | 0.558 |
| edm - rap | 5 | 0.536 | 0.123 | 33661.857 | 4.374 | < .001 |
| edm - rock | 5 | 0.188 | 0.123 | 33019.072 | 1.52 | 0.651 |
| latin - pop | 5 | -0.46 | 0.14 | 33569.531 | -3.275 | 0.014 |
| latin - r&b | 5 | -0.237 | 0.143 | 33491.514 | -1.664 | 0.556 |
| latin - rap | 5 | 0.499 | 0.145 | 33863.974 | 3.449 | 0.007 |
| latin - rock | 5 | 0.15 | 0.145 | 33394.508 | 1.033 | 0.907 |
| pop - r&b | 5 | 0.222 | 0.089 | 33453.686 | 2.505 | 0.123 |
| pop - rap | 5 | 0.958 | 0.092 | 34387.381 | 10.447 | < .001 |
| pop - rock | 5 | 0.61 | 0.093 | 33219.85 | 6.57 | < .001 |
| r&b - rap | 5 | 0.736 | 0.095 | 34149.105 | 7.729 | < .001 |
| r&b - rock | 5 | 0.387 | 0.096 | 33074.928 | 4.025 | 0.001 |
| rap - rock | 5 | -0.349 | 0.099 | 33887.454 | -3.52 | 0.006 |

Table 4. Pairwise comparisons of Plot Progression word rates within segments between genres (larger dataset)

| contrast | Segment | estimate | | SE | | df | | t.ratio | | p.value | |  |
| --- | --- | --- | --- | --- | --- | --- | --- | --- | --- | --- | --- | --- |
| edm - latin | 1 | | 2.288 | | 0.543 | | 26694.596 | | 4.215 | | < .001 | |
| edm - pop | 1 | | -0.413 | | 0.393 | | 26551.347 | | -1.05 | | 0.901 | |
| edm - r&b | 1 | | -0.91 | | 0.403 | | 26501.8 | | -2.26 | | 0.211 | |
| edm - rap | 1 | | 7.832 | | 0.41 | | 26851.639 | | 19.122 | | < .001 | |
| edm - rock | 1 | | 1.945 | | 0.413 | | 26381.556 | | 4.708 | | < .001 | |
| latin - pop | 1 | | -2.701 | | 0.469 | | 26820.2 | | -5.759 | | < .001 | |
| latin - r&b | 1 | | -3.198 | | 0.477 | | 26775.473 | | -6.705 | | < .001 | |
| latin - rap | 1 | | 5.544 | | 0.483 | | 27023.252 | | 11.487 | | < .001 | |
| latin - rock | 1 | | -0.343 | | 0.486 | | 26677.788 | | -0.706 | | 0.981 | |
| pop - r&b | 1 | | -0.497 | | 0.296 | | 26650.608 | | -1.679 | | 0.546 | |
| pop - rap | 1 | | 8.245 | | 0.305 | | 27279.139 | | 27.011 | | < .001 | |
| pop - rock | 1 | | 2.358 | | 0.31 | | 26423.292 | | 7.609 | | < .001 | |
| r&b - rap | 1 | | 8.742 | | 0.317 | | 27141.716 | | 27.544 | | < .001 | |
| r&b - rock | 1 | | 2.855 | | 0.322 | | 26355.414 | | 8.871 | | < .001 | |
| rap - rock | 1 | | -5.887 | | 0.33 | | 26899.951 | | -17.831 | | < .001 | |
| edm - latin | 2 | | 2.103 | | 0.539 | | 26116.775 | | 3.901 | | 0.001 | |
| edm - pop | 2 | | -0.211 | | 0.391 | | 25996.438 | | -0.541 | | 0.994 | |
| edm - r&b | 2 | | -0.996 | | 0.4 | | 25943.037 | | -2.488 | | 0.128 | |
| edm - rap | 2 | | 7.911 | | 0.407 | | 26284.557 | | 19.444 | | < .001 | |
| edm - rock | 2 | | 2.657 | | 0.41 | | 25860.128 | | 6.474 | | < .001 | |
| latin - pop | 2 | | -2.314 | | 0.466 | | 26292.597 | | -4.965 | | < .001 | |
| latin - r&b | 2 | | -3.098 | | 0.474 | | 26244.202 | | -6.537 | | < .001 | |
| latin - rap | 2 | | 5.808 | | 0.48 | | 26485.427 | | 12.108 | | < .001 | |
| latin - rock | 2 | | 0.554 | | 0.483 | | 26172.664 | | 1.148 | | 0.861 | |
| pop - r&b | 2 | | -0.784 | | 0.295 | | 26233.665 | | -2.661 | | 0.083 | |
| pop - rap | 2 | | 8.122 | | 0.304 | | 26840.237 | | 26.74 | | < .001 | |
| pop - rock | 2 | | 2.868 | | 0.309 | | 26059.924 | | 9.297 | | < .001 | |
| r&b - rap | 2 | | 8.906 | | 0.316 | | 26687.705 | | 28.208 | | < .001 | |
| r&b - rock | 2 | | 3.652 | | 0.32 | | 25971.823 | | 11.402 | | < .001 | |
| rap - rock | 2 | | -5.254 | | 0.329 | | 26496.513 | | -15.987 | | < .001 | |
| edm - latin | 3 | | 1.178 | | 0.538 | | 25909.38 | | 2.19 | | 0.242 | |
| edm - pop | 3 | | -0.376 | | 0.39 | | 25811.327 | | -0.963 | | 0.929 | |
| edm - r&b | 3 | | -0.749 | | 0.399 | | 25734.187 | | -1.876 | | 0.417 | |
| edm - rap | 3 | | 8.208 | | 0.406 | | 26109.866 | | 20.218 | | < .001 | |
| edm - rock | 3 | | 2.705 | | 0.41 | | 25697.072 | | 6.605 | | < .001 | |
| latin - pop | 3 | | -1.553 | | 0.465 | | 26094.247 | | -3.34 | | 0.011 | |
| latin - r&b | 3 | | -1.927 | | 0.473 | | 26029.297 | | -4.075 | | 0.001 | |
| latin - rap | 3 | | 7.03 | | 0.479 | | 26295.255 | | 14.689 | | < .001 | |
| latin - rock | 3 | | 1.528 | | 0.482 | | 25991.292 | | 3.172 | | 0.019 | |
| pop - r&b | 3 | | -0.373 | | 0.294 | | 26046.251 | | -1.269 | | 0.802 | |
| pop - rap | 3 | | 8.583 | | 0.303 | | 26713.635 | | 28.3 | | < .001 | |
| pop - rock | 3 | | 3.081 | | 0.308 | | 25951.906 | | 10 | | < .001 | |
| r&b - rap | 3 | | 8.957 | | 0.315 | | 26517.969 | | 28.425 | | < .001 | |
| r&b - rock | 3 | | 3.454 | | 0.32 | | 25821.059 | | 10.804 | | < .001 | |
| rap - rock | 3 | | -5.502 | | 0.328 | | 26395.888 | | -16.763 | | < .001 | |
| edm - latin | 4 | | 2.5 | | 0.539 | | 26071.288 | | 4.64 | | < .001 | |
| edm - pop | 4 | | -0.324 | | 0.391 | | 26025.501 | | -0.829 | | 0.962 | |
| edm - r&b | 4 | | -0.008 | | 0.4 | | 25947.478 | | -0.019 | | 1 | |
| edm - rap | 4 | | 8.254 | | 0.407 | | 26319.266 | | 20.279 | | < .001 | |
| edm - rock | 4 | | 2.352 | | 0.411 | | 25880.892 | | 5.73 | | < .001 | |
| latin - pop | 4 | | -2.824 | | 0.466 | | 26233.514 | | -6.062 | | < .001 | |
| latin - r&b | 4 | | -2.507 | | 0.474 | | 26170.459 | | -5.294 | | < .001 | |
| latin - rap | 4 | | 5.754 | | 0.479 | | 26435.031 | | 12.003 | | < .001 | |
| latin - rock | 4 | | -0.147 | | 0.482 | | 26113.692 | | -0.306 | | 1 | |
| pop - r&b | 4 | | 0.317 | | 0.295 | | 26246.772 | | 1.074 | | 0.892 | |
| pop - rap | 4 | | 8.578 | | 0.304 | | 26907.794 | | 28.219 | | < .001 | |
| pop - rock | 4 | | 2.676 | | 0.309 | | 26101.256 | | 8.67 | | < .001 | |
| r&b - rap | 4 | | 8.261 | | 0.316 | | 26712.212 | | 26.159 | | < .001 | |
| r&b - rock | 4 | | 2.36 | | 0.32 | | 25973.809 | | 7.367 | | < .001 | |
| rap - rock | 4 | | -5.901 | | 0.329 | | 26545.17 | | -17.948 | | < .001 | |
| edm - latin | 5 | | 2.532 | | 0.542 | | 26599.473 | | 4.671 | | < .001 | |
| edm - pop | 5 | | -0.486 | | 0.393 | | 26497.828 | | -1.236 | | 0.819 | |
| edm - r&b | 5 | | -0.859 | | 0.402 | | 26420.776 | | -2.135 | | 0.269 | |
| edm - rap | 5 | | 7.199 | | 0.409 | | 26836.137 | | 17.582 | | < .001 | |
| edm - rock | 5 | | 3.678 | | 0.413 | | 26330.482 | | 8.91 | | < .001 | |
| latin - pop | 5 | | -3.018 | | 0.469 | | 26766.735 | | -6.438 | | < .001 | |
| latin - r&b | 5 | | -3.392 | | 0.477 | | 26702.319 | | -7.116 | | < .001 | |
| latin - rap | 5 | | 4.667 | | 0.483 | | 26997.187 | | 9.671 | | < .001 | |
| latin - rock | 5 | | 1.145 | | 0.485 | | 26626.156 | | 2.36 | | 0.171 | |
| pop - r&b | 5 | | -0.374 | | 0.296 | | 26685.878 | | -1.26 | | 0.807 | |
| pop - rap | 5 | | 7.685 | | 0.306 | | 27427.588 | | 25.134 | | < .001 | |
| pop - rock | 5 | | 4.164 | | 0.31 | | 26501.333 | | 13.424 | | < .001 | |
| r&b - rap | 5 | | 8.059 | | 0.318 | | 27229.856 | | 25.366 | | < .001 | |
| r&b - rock | 5 | | 4.537 | | 0.322 | | 26380.736 | | 14.094 | | < .001 | |
| rap - rock | 5 | | -3.522 | | 0.331 | | 27021.845 | | -10.651 | | < .001 | |

Table 5. Pairwise comparisons of Staging word rates within segments between genres (larger dataset)

| contrast | Segment | estimate | SE | df | t.ratio | p.value |
| --- | --- | --- | --- | --- | --- | --- |
| edm - latin | 1 | 0.311 | 0.321 | 31002.714 | 0.97 | 0.927 |
| edm - pop | 1 | 0.008 | 0.233 | 30822.748 | 0.033 | 1 |
| edm - r&b | 1 | 0.934 | 0.238 | 30766.513 | 3.922 | 0.001 |
| edm - rap | 1 | -0.626 | 0.242 | 31178.461 | -2.583 | 0.101 |
| edm - rock | 1 | -1.821 | 0.244 | 30620.238 | -7.459 | < .001 |
| latin - pop | 1 | -0.304 | 0.278 | 31149.251 | -1.095 | 0.884 |
| latin - r&b | 1 | 0.623 | 0.282 | 31097.914 | 2.207 | 0.234 |
| latin - rap | 1 | -0.938 | 0.286 | 31388.731 | -3.282 | 0.013 |
| latin - rock | 1 | -2.133 | 0.287 | 30979.494 | -7.425 | < .001 |
| pop - r&b | 1 | 0.927 | 0.175 | 30931.231 | 5.289 | < .001 |
| pop - rap | 1 | -0.634 | 0.181 | 31669.117 | -3.505 | 0.006 |
| pop - rock | 1 | -1.829 | 0.183 | 30656.494 | -9.983 | < .001 |
| r&b - rap | 1 | -1.56 | 0.188 | 31512.658 | -8.303 | < .001 |
| r&b - rock | 1 | -2.756 | 0.19 | 30580.873 | -14.484 | < .001 |
| rap - rock | 1 | -1.195 | 0.195 | 31222.899 | -6.117 | < .001 |
| edm - latin | 2 | 0.119 | 0.318 | 30286.67 | 0.373 | 0.999 |
| edm - pop | 2 | -0.065 | 0.231 | 30135.966 | -0.281 | 1 |
| edm - r&b | 2 | 0.129 | 0.236 | 30076.554 | 0.548 | 0.994 |
| edm - rap | 2 | -1.801 | 0.24 | 30477.316 | -7.49 | < .001 |
| edm - rock | 2 | -2.401 | 0.242 | 29977.113 | -9.908 | < .001 |
| latin - pop | 2 | -0.184 | 0.276 | 30494.231 | -0.666 | 0.986 |
| latin - r&b | 2 | 0.011 | 0.28 | 30439.597 | 0.038 | 1 |
| latin - rap | 2 | -1.92 | 0.284 | 30721.629 | -6.769 | < .001 |
| latin - rock | 2 | -2.52 | 0.285 | 30354.003 | -8.835 | < .001 |
| pop - r&b | 2 | 0.194 | 0.174 | 30416.189 | 1.116 | 0.875 |
| pop - rap | 2 | -1.736 | 0.18 | 31124.274 | -9.66 | < .001 |
| pop - rock | 2 | -2.337 | 0.182 | 30209.162 | -12.82 | < .001 |
| r&b - rap | 2 | -1.931 | 0.187 | 30952.126 | -10.337 | < .001 |
| r&b - rock | 2 | -2.531 | 0.189 | 30111.012 | -13.376 | < .001 |
| rap - rock | 2 | -0.6 | 0.194 | 30725.773 | -3.089 | 0.025 |
| edm - latin | 3 | 0.711 | 0.318 | 30031.37 | 2.238 | 0.22 |
| edm - pop | 3 | 0.358 | 0.23 | 29912.025 | 1.553 | 0.63 |
| edm - r&b | 3 | 0.637 | 0.236 | 29823.411 | 2.705 | 0.074 |
| edm - rap | 3 | -1.187 | 0.24 | 30264.983 | -4.949 | < .001 |
| edm - rock | 3 | -1.901 | 0.242 | 29779.669 | -7.863 | < .001 |
| latin - pop | 3 | -0.353 | 0.275 | 30248.623 | -1.286 | 0.793 |
| latin - r&b | 3 | -0.073 | 0.279 | 30173.868 | -0.262 | 1 |
| latin - rap | 3 | -1.898 | 0.283 | 30485.49 | -6.709 | < .001 |
| latin - rock | 3 | -2.612 | 0.285 | 30129.113 | -9.18 | < .001 |
| pop - r&b | 3 | 0.28 | 0.174 | 30189.255 | 1.611 | 0.591 |
| pop - rap | 3 | -1.545 | 0.179 | 30969.272 | -8.608 | < .001 |
| pop - rock | 3 | -2.259 | 0.182 | 30078.559 | -12.411 | < .001 |
| r&b - rap | 3 | -1.825 | 0.186 | 30744.844 | -9.792 | < .001 |
| r&b - rock | 3 | -2.539 | 0.189 | 29928.078 | -13.446 | < .001 |
| rap - rock | 3 | -0.714 | 0.194 | 30602.088 | -3.679 | 0.003 |
| edm - latin | 4 | 0.194 | 0.318 | 30230.077 | 0.608 | 0.991 |
| edm - pop | 4 | 0.074 | 0.231 | 30171.482 | 0.319 | 1 |
| edm - r&b | 4 | 0.143 | 0.236 | 30081.926 | 0.605 | 0.991 |
| edm - rap | 4 | -2.088 | 0.241 | 30521.545 | -8.679 | < .001 |
| edm - rock | 4 | -2.05 | 0.242 | 30002.76 | -8.455 | < .001 |
| latin - pop | 4 | -0.12 | 0.275 | 30420.212 | -0.435 | 0.998 |
| latin - r&b | 4 | -0.051 | 0.28 | 30347.681 | -0.18 | 1 |
| latin - rap | 4 | -2.282 | 0.283 | 30659.848 | -8.05 | < .001 |
| latin - rock | 4 | -2.243 | 0.285 | 30280.333 | -7.871 | < .001 |
| pop - r&b | 4 | 0.069 | 0.174 | 30430.556 | 0.398 | 0.999 |
| pop - rap | 4 | -2.162 | 0.18 | 31208.137 | -12.017 | < .001 |
| pop - rock | 4 | -2.123 | 0.182 | 30258.682 | -11.643 | < .001 |
| r&b - rap | 4 | -2.231 | 0.187 | 30983.697 | -11.942 | < .001 |
| r&b - rock | 4 | -2.193 | 0.189 | 30112.469 | -11.589 | < .001 |
| rap - rock | 4 | 0.038 | 0.194 | 30787.051 | 0.197 | 1 |
| edm - latin | 5 | 0.15 | 0.321 | 30880.003 | 0.467 | 0.997 |
| edm - pop | 5 | -0.074 | 0.232 | 30755.014 | -0.317 | 1 |
| edm - r&b | 5 | 0.219 | 0.238 | 30665.926 | 0.919 | 0.942 |
| edm - rap | 5 | -1.849 | 0.242 | 31160.807 | -7.631 | < .001 |
| edm - rock | 5 | -2.416 | 0.244 | 30557.552 | -9.902 | < .001 |
| latin - pop | 5 | -0.224 | 0.277 | 31075.356 | -0.806 | 0.967 |
| latin - r&b | 5 | 0.069 | 0.282 | 31000.794 | 0.245 | 1 |
| latin - rap | 5 | -1.999 | 0.286 | 31351.19 | -6.997 | < .001 |
| latin - rock | 5 | -2.566 | 0.287 | 30909.78 | -8.937 | < .001 |
| pop - r&b | 5 | 0.292 | 0.175 | 30971.834 | 1.668 | 0.553 |
| pop - rap | 5 | -1.775 | 0.181 | 31851.359 | -9.8 | < .001 |
| pop - rock | 5 | -2.342 | 0.183 | 30751.981 | -12.771 | < .001 |
| r&b - rap | 5 | -2.068 | 0.188 | 31623.101 | -10.99 | < .001 |
| r&b - rock | 5 | -2.635 | 0.19 | 30613.047 | -13.844 | < .001 |
| rap - rock | 5 | -0.567 | 0.196 | 31376.386 | -2.897 | 0.044 |

## Pairwise comparisons of word rates across segments within genre

Table 6. Pairwise comparisons of Cognitive Tension word rates within genres between segments (larger dataset)

| contrast | genre | estimate | SE | df | t.ratio | p.value |
| --- | --- | --- | --- | --- | --- | --- |
| Segment1 - Segment2 | edm | 0.386 | 0.103 | 48696.654 | 3.76 | 0.002 |
| Segment1 - Segment3 | edm | 0.257 | 0.103 | 48756.508 | 2.51 | 0.088 |
| Segment1 - Segment4 | edm | 0.583 | 0.103 | 48862.315 | 5.661 | < .001 |
| Segment1 - Segment5 | edm | 0.889 | 0.104 | 48965.93 | 8.56 | < .001 |
| Segment2 - Segment3 | edm | -0.129 | 0.102 | 48660.933 | -1.266 | 0.712 |
| Segment2 - Segment4 | edm | 0.197 | 0.102 | 48764.351 | 1.931 | 0.301 |
| Segment2 - Segment5 | edm | 0.503 | 0.103 | 48850.498 | 4.889 | < .001 |
| Segment3 - Segment4 | edm | 0.326 | 0.102 | 48673.845 | 3.204 | 0.012 |
| Segment3 - Segment5 | edm | 0.631 | 0.102 | 48758.291 | 6.164 | < .001 |
| Segment4 - Segment5 | edm | 0.306 | 0.103 | 48685.453 | 2.981 | 0.024 |
| Segment1 - Segment2 | latin | 0.112 | 0.129 | 48723.053 | 0.865 | 0.91 |
| Segment1 - Segment3 | latin | 0 | 0.129 | 48914.868 | 0.002 | 1 |
| Segment1 - Segment4 | latin | 0.288 | 0.13 | 49060.501 | 2.218 | 0.173 |
| Segment1 - Segment5 | latin | 0.575 | 0.131 | 49103.223 | 4.394 | < .001 |
| Segment2 - Segment3 | latin | -0.112 | 0.128 | 48789.776 | -0.87 | 0.908 |
| Segment2 - Segment4 | latin | 0.176 | 0.129 | 48933.726 | 1.369 | 0.648 |
| Segment2 - Segment5 | latin | 0.463 | 0.13 | 48995.442 | 3.569 | 0.003 |
| Segment3 - Segment4 | latin | 0.288 | 0.128 | 48723.188 | 2.249 | 0.162 |
| Segment3 - Segment5 | latin | 0.574 | 0.129 | 48801.123 | 4.453 | < .001 |
| Segment4 - Segment5 | latin | 0.287 | 0.129 | 48677.804 | 2.224 | 0.171 |
| Segment1 - Segment2 | pop | 0.255 | 0.061 | 48799.411 | 4.172 | < .001 |
| Segment1 - Segment3 | pop | 0.137 | 0.061 | 48960.926 | 2.246 | 0.163 |
| Segment1 - Segment4 | pop | 0.315 | 0.062 | 49208.151 | 5.126 | < .001 |
| Segment1 - Segment5 | pop | 0.614 | 0.062 | 49423.867 | 9.903 | < .001 |
| Segment2 - Segment3 | pop | -0.117 | 0.061 | 48775.708 | -1.933 | 0.3 |
| Segment2 - Segment4 | pop | 0.061 | 0.061 | 49033.486 | 0.993 | 0.859 |
| Segment2 - Segment5 | pop | 0.36 | 0.062 | 49198.023 | 5.846 | < .001 |
| Segment3 - Segment4 | pop | 0.178 | 0.061 | 48780.49 | 2.93 | 0.028 |
| Segment3 - Segment5 | pop | 0.477 | 0.061 | 48948.306 | 7.797 | < .001 |
| Segment4 - Segment5 | pop | 0.299 | 0.061 | 48771.476 | 4.893 | < .001 |
| Segment1 - Segment2 | r&b | 0.046 | 0.066 | 48683.193 | 0.692 | 0.958 |
| Segment1 - Segment3 | r&b | 0.237 | 0.066 | 48829.435 | 3.569 | 0.003 |
| Segment1 - Segment4 | r&b | 0.448 | 0.067 | 49017.982 | 6.714 | < .001 |
| Segment1 - Segment5 | r&b | 0.739 | 0.067 | 49140.575 | 10.991 | < .001 |
| Segment2 - Segment3 | r&b | 0.191 | 0.066 | 48746.812 | 2.896 | 0.031 |
| Segment2 - Segment4 | r&b | 0.402 | 0.066 | 48937.758 | 6.066 | < .001 |
| Segment2 - Segment5 | r&b | 0.693 | 0.067 | 49028.003 | 10.384 | < .001 |
| Segment3 - Segment4 | r&b | 0.211 | 0.066 | 48730.487 | 3.205 | 0.012 |
| Segment3 - Segment5 | r&b | 0.502 | 0.066 | 48819.986 | 7.569 | < .001 |
| Segment4 - Segment5 | r&b | 0.291 | 0.066 | 48690.179 | 4.387 | < .001 |
| Segment1 - Segment2 | rap | 0.009 | 0.071 | 48825.906 | 0.127 | 1 |
| Segment1 - Segment3 | rap | -0.075 | 0.071 | 49200.616 | -1.047 | 0.834 |
| Segment1 - Segment4 | rap | 0.02 | 0.072 | 49637.541 | 0.273 | 0.999 |
| Segment1 - Segment5 | rap | 0.005 | 0.073 | 49911.704 | 0.073 | 1 |
| Segment2 - Segment3 | rap | -0.084 | 0.071 | 48964.483 | -1.183 | 0.761 |
| Segment2 - Segment4 | rap | 0.011 | 0.071 | 49411.366 | 0.149 | 1 |
| Segment2 - Segment5 | rap | -0.004 | 0.072 | 49666.752 | -0.052 | 1 |
| Segment3 - Segment4 | rap | 0.094 | 0.071 | 48992.863 | 1.331 | 0.672 |
| Segment3 - Segment5 | rap | 0.08 | 0.071 | 49269.619 | 1.117 | 0.797 |
| Segment4 - Segment5 | rap | -0.014 | 0.071 | 48868.708 | -0.201 | 1 |
| Segment1 - Segment2 | rock | 0.194 | 0.072 | 48662.344 | 2.706 | 0.053 |
| Segment1 - Segment3 | rock | 0.239 | 0.072 | 48749.713 | 3.329 | 0.008 |
| Segment1 - Segment4 | rock | 0.372 | 0.072 | 48862.516 | 5.167 | < .001 |
| Segment1 - Segment5 | rock | 0.784 | 0.072 | 48962.133 | 10.828 | < .001 |
| Segment2 - Segment3 | rock | 0.045 | 0.071 | 48684.865 | 0.628 | 0.971 |
| Segment2 - Segment4 | rock | 0.178 | 0.072 | 48800.257 | 2.485 | 0.094 |
| Segment2 - Segment5 | rock | 0.59 | 0.072 | 48865.118 | 8.2 | < .001 |
| Segment3 - Segment4 | rock | 0.133 | 0.071 | 48679.902 | 1.865 | 0.337 |
| Segment3 - Segment5 | rock | 0.545 | 0.072 | 48748.665 | 7.6 | < .001 |
| Segment4 - Segment5 | rock | 0.412 | 0.072 | 48671.463 | 5.747 | < .001 |

Table 7. Pairwise comparisons of Plot Progression word rates within genres between segments (larger dataset)

| contrast | genre | estimate | SE | df | t.ratio | p.value |
| --- | --- | --- | --- | --- | --- | --- |
| Segment1 - Segment2 | edm | -0.79 | 0.303 | 48655.862 | -2.611 | 0.068 |
| Segment1 - Segment3 | edm | -0.781 | 0.302 | 48698.28 | -2.582 | 0.074 |
| Segment1 - Segment4 | edm | -0.912 | 0.304 | 48772.53 | -3.003 | 0.022 |
| Segment1 - Segment5 | edm | -0.137 | 0.306 | 48846.111 | -0.447 | 0.992 |
| Segment2 - Segment3 | edm | 0.009 | 0.3 | 48629.975 | 0.032 | 1 |
| Segment2 - Segment4 | edm | -0.122 | 0.301 | 48702.481 | -0.405 | 0.994 |
| Segment2 - Segment5 | edm | 0.653 | 0.303 | 48764.403 | 2.154 | 0.197 |
| Segment3 - Segment4 | edm | -0.131 | 0.3 | 48639.543 | -0.438 | 0.992 |
| Segment3 - Segment5 | edm | 0.644 | 0.302 | 48700.22 | 2.132 | 0.206 |
| Segment4 - Segment5 | edm | 0.775 | 0.302 | 48648.157 | 2.565 | 0.077 |
| Segment1 - Segment2 | latin | -0.975 | 0.381 | 48680.673 | -2.558 | 0.078 |
| Segment1 - Segment3 | latin | -1.891 | 0.381 | 48823.263 | -4.957 | < .001 |
| Segment1 - Segment4 | latin | -0.7 | 0.383 | 48929.588 | -1.829 | 0.357 |
| Segment1 - Segment5 | latin | 0.108 | 0.386 | 48957.964 | 0.279 | 0.999 |
| Segment2 - Segment3 | latin | -0.916 | 0.378 | 48727.489 | -2.423 | 0.109 |
| Segment2 - Segment4 | latin | 0.275 | 0.379 | 48832.594 | 0.724 | 0.951 |
| Segment2 - Segment5 | latin | 1.083 | 0.383 | 48874.679 | 2.83 | 0.037 |
| Segment3 - Segment4 | latin | 1.19 | 0.377 | 48677.607 | 3.156 | 0.014 |
| Segment3 - Segment5 | latin | 1.999 | 0.38 | 48732.311 | 5.254 | < .001 |
| Segment4 - Segment5 | latin | 0.808 | 0.38 | 48641.268 | 2.126 | 0.209 |
| Segment1 - Segment2 | pop | -0.588 | 0.18 | 48743.36 | -3.27 | 0.009 |
| Segment1 - Segment3 | pop | -0.743 | 0.18 | 48861.099 | -4.123 | < .001 |
| Segment1 - Segment4 | pop | -0.823 | 0.181 | 49037.499 | -4.536 | < .001 |
| Segment1 - Segment5 | pop | -0.21 | 0.183 | 49199.587 | -1.144 | 0.783 |
| Segment2 - Segment3 | pop | -0.155 | 0.179 | 48715.77 | -0.865 | 0.91 |
| Segment2 - Segment4 | pop | -0.235 | 0.18 | 48899.488 | -1.303 | 0.689 |
| Segment2 - Segment5 | pop | 0.379 | 0.182 | 49027.758 | 2.087 | 0.226 |
| Segment3 - Segment4 | pop | -0.08 | 0.179 | 48718.018 | -0.446 | 0.992 |
| Segment3 - Segment5 | pop | 0.534 | 0.18 | 48848.739 | 2.957 | 0.026 |
| Segment4 - Segment5 | pop | 0.614 | 0.18 | 48719.431 | 3.405 | 0.006 |
| Segment1 - Segment2 | r&b | -0.875 | 0.196 | 48646.028 | -4.477 | < .001 |
| Segment1 - Segment3 | r&b | -0.619 | 0.196 | 48752.354 | -3.166 | 0.013 |
| Segment1 - Segment4 | r&b | -0.009 | 0.197 | 48885.83 | -0.048 | 1 |
| Segment1 - Segment5 | r&b | -0.086 | 0.198 | 48972.868 | -0.433 | 0.993 |
| Segment2 - Segment3 | r&b | 0.256 | 0.194 | 48693.318 | 1.318 | 0.68 |
| Segment2 - Segment4 | r&b | 0.866 | 0.195 | 48828.407 | 4.432 | < .001 |
| Segment2 - Segment5 | r&b | 0.79 | 0.197 | 48893.935 | 4.013 | 0.001 |
| Segment3 - Segment4 | r&b | 0.61 | 0.194 | 48681.049 | 3.141 | 0.015 |
| Segment3 - Segment5 | r&b | 0.533 | 0.196 | 48745.933 | 2.729 | 0.05 |
| Segment4 - Segment5 | r&b | -0.076 | 0.195 | 48651.966 | -0.391 | 0.995 |
| Segment1 - Segment2 | rap | -0.712 | 0.209 | 48767.002 | -3.404 | 0.006 |
| Segment1 - Segment3 | rap | -0.405 | 0.21 | 49047.959 | -1.927 | 0.303 |
| Segment1 - Segment4 | rap | -0.49 | 0.212 | 49371.01 | -2.312 | 0.141 |
| Segment1 - Segment5 | rap | -0.769 | 0.214 | 49588.43 | -3.588 | 0.003 |
| Segment2 - Segment3 | rap | 0.307 | 0.208 | 48858.957 | 1.473 | 0.58 |
| Segment2 - Segment4 | rap | 0.221 | 0.21 | 49188.871 | 1.052 | 0.831 |
| Segment2 - Segment5 | rap | -0.058 | 0.212 | 49395.2 | -0.272 | 0.999 |
| Segment3 - Segment4 | rap | -0.086 | 0.209 | 48880.152 | -0.41 | 0.994 |
| Segment3 - Segment5 | rap | -0.365 | 0.211 | 49102.739 | -1.73 | 0.416 |
| Segment4 - Segment5 | rap | -0.279 | 0.21 | 48801.826 | -1.33 | 0.673 |
| Segment1 - Segment2 | rock | -0.078 | 0.211 | 48629.51 | -0.37 | 0.996 |
| Segment1 - Segment3 | rock | -0.02 | 0.211 | 48692.366 | -0.095 | 1 |
| Segment1 - Segment4 | rock | -0.505 | 0.212 | 48771.801 | -2.38 | 0.121 |
| Segment1 - Segment5 | rock | 1.596 | 0.213 | 48840.707 | 7.478 | < .001 |
| Segment2 - Segment3 | rock | 0.058 | 0.21 | 48647.969 | 0.276 | 0.999 |
| Segment2 - Segment4 | rock | -0.427 | 0.211 | 48729.601 | -2.023 | 0.255 |
| Segment2 - Segment5 | rock | 1.674 | 0.212 | 48775.33 | 7.892 | < .001 |
| Segment3 - Segment4 | rock | -0.485 | 0.21 | 48644.667 | -2.305 | 0.143 |
| Segment3 - Segment5 | rock | 1.616 | 0.211 | 48692.958 | 7.642 | < .001 |
| Segment4 - Segment5 | rock | 2.101 | 0.211 | 48636.907 | 9.937 | < .001 |

Table 8. Pairwise comparisons of Staging word rates within genres between segments (larger dataset)

| contrast | genre | estimate | SE | df | t.ratio | p.value |
| --- | --- | --- | --- | --- | --- | --- |
| Segment1 - Segment2 | edm | 0.92 | 0.195 | 48681.39 | 4.714 | < .001 |
| Segment1 - Segment3 | edm | 0.448 | 0.195 | 48734.911 | 2.298 | 0.145 |
| Segment1 - Segment4 | edm | 1.114 | 0.196 | 48829.174 | 5.69 | < .001 |
| Segment1 - Segment5 | edm | 1.85 | 0.197 | 48921.778 | 9.378 | < .001 |
| Segment2 - Segment3 | edm | -0.472 | 0.193 | 48649.301 | -2.443 | 0.104 |
| Segment2 - Segment4 | edm | 0.194 | 0.194 | 48741.401 | 1.002 | 0.855 |
| Segment2 - Segment5 | edm | 0.931 | 0.195 | 48818.722 | 4.763 | < .001 |
| Segment3 - Segment4 | edm | 0.666 | 0.193 | 48661.039 | 3.448 | 0.005 |
| Segment3 - Segment5 | edm | 1.402 | 0.195 | 48736.818 | 7.206 | < .001 |
| Segment4 - Segment5 | edm | 0.737 | 0.195 | 48671.482 | 3.78 | 0.001 |
| Segment1 - Segment2 | latin | 0.727 | 0.246 | 48707.578 | 2.959 | 0.026 |
| Segment1 - Segment3 | latin | 0.847 | 0.246 | 48882.128 | 3.448 | 0.005 |
| Segment1 - Segment4 | latin | 0.996 | 0.247 | 49013.76 | 4.038 | 0.001 |
| Segment1 - Segment5 | latin | 1.689 | 0.249 | 49051.096 | 6.794 | < .001 |
| Segment2 - Segment3 | latin | 0.12 | 0.244 | 48767.344 | 0.494 | 0.988 |
| Segment2 - Segment4 | latin | 0.269 | 0.244 | 48897.459 | 1.101 | 0.806 |
| Segment2 - Segment5 | latin | 0.962 | 0.246 | 48951.891 | 3.903 | 0.001 |
| Segment3 - Segment4 | latin | 0.149 | 0.243 | 48706.572 | 0.612 | 0.973 |
| Segment3 - Segment5 | latin | 0.842 | 0.245 | 48776.014 | 3.434 | 0.005 |
| Segment4 - Segment5 | latin | 0.693 | 0.245 | 48664.078 | 2.828 | 0.038 |
| Segment1 - Segment2 | pop | 0.847 | 0.116 | 48779.411 | 7.306 | < .001 |
| Segment1 - Segment3 | pop | 0.798 | 0.116 | 48925.334 | 6.87 | < .001 |
| Segment1 - Segment4 | pop | 1.18 | 0.117 | 49146.883 | 10.095 | < .001 |
| Segment1 - Segment5 | pop | 1.769 | 0.118 | 49343.388 | 15.003 | < .001 |
| Segment2 - Segment3 | pop | -0.049 | 0.115 | 48753.914 | -0.427 | 0.993 |
| Segment2 - Segment4 | pop | 0.333 | 0.116 | 48984.832 | 2.868 | 0.034 |
| Segment2 - Segment5 | pop | 0.922 | 0.117 | 49136.725 | 7.884 | < .001 |
| Segment3 - Segment4 | pop | 0.382 | 0.115 | 48757.708 | 3.311 | 0.008 |
| Segment3 - Segment5 | pop | 0.971 | 0.116 | 48912.596 | 8.353 | < .001 |
| Segment4 - Segment5 | pop | 0.589 | 0.116 | 48752.675 | 5.072 | < .001 |
| Segment1 - Segment2 | r&b | 0.115 | 0.126 | 48669.233 | 0.911 | 0.893 |
| Segment1 - Segment3 | r&b | 0.151 | 0.126 | 48801.22 | 1.199 | 0.752 |
| Segment1 - Segment4 | r&b | 0.323 | 0.127 | 48969.712 | 2.546 | 0.081 |
| Segment1 - Segment5 | r&b | 1.135 | 0.128 | 49079.248 | 8.887 | < .001 |
| Segment2 - Segment3 | r&b | 0.036 | 0.125 | 48727.219 | 0.29 | 0.998 |
| Segment2 - Segment4 | r&b | 0.208 | 0.126 | 48897.814 | 1.651 | 0.465 |
| Segment2 - Segment5 | r&b | 1.02 | 0.127 | 48979.09 | 8.047 | < .001 |
| Segment3 - Segment4 | r&b | 0.172 | 0.125 | 48712.302 | 1.371 | 0.646 |
| Segment3 - Segment5 | r&b | 0.984 | 0.126 | 48792.855 | 7.809 | < .001 |
| Segment4 - Segment5 | r&b | 0.812 | 0.126 | 48675.889 | 6.45 | < .001 |
| Segment1 - Segment2 | rap | -0.255 | 0.135 | 48805.141 | -1.896 | 0.319 |
| Segment1 - Segment3 | rap | -0.113 | 0.135 | 49147.137 | -0.835 | 0.92 |
| Segment1 - Segment4 | rap | -0.348 | 0.137 | 49543.888 | -2.55 | 0.08 |
| Segment1 - Segment5 | rap | 0.628 | 0.138 | 49798.511 | 4.548 | < .001 |
| Segment2 - Segment3 | rap | 0.142 | 0.134 | 48926.986 | 1.062 | 0.826 |
| Segment2 - Segment4 | rap | -0.093 | 0.135 | 49332.576 | -0.685 | 0.96 |
| Segment2 - Segment5 | rap | 0.883 | 0.137 | 49571.433 | 6.456 | < .001 |
| Segment3 - Segment4 | rap | -0.235 | 0.134 | 48952.864 | -1.75 | 0.403 |
| Segment3 - Segment5 | rap | 0.741 | 0.136 | 49211.358 | 5.455 | < .001 |
| Segment4 - Segment5 | rap | 0.976 | 0.135 | 48845.304 | 7.216 | < .001 |
| Segment1 - Segment2 | rock | 0.34 | 0.136 | 48649.875 | 2.496 | 0.092 |
| Segment1 - Segment3 | rock | 0.368 | 0.136 | 48728.416 | 2.704 | 0.053 |
| Segment1 - Segment4 | rock | 0.886 | 0.137 | 48829.023 | 6.481 | < .001 |
| Segment1 - Segment5 | rock | 1.256 | 0.138 | 48917.244 | 9.131 | < .001 |
| Segment2 - Segment3 | rock | 0.029 | 0.135 | 48671.16 | 0.212 | 1 |
| Segment2 - Segment4 | rock | 0.546 | 0.136 | 48774.246 | 4.017 | 0.001 |
| Segment2 - Segment5 | rock | 0.916 | 0.137 | 48832.075 | 6.703 | < .001 |
| Segment3 - Segment4 | rock | 0.517 | 0.135 | 48666.824 | 3.817 | 0.001 |
| Segment3 - Segment5 | rock | 0.888 | 0.136 | 48728.045 | 6.512 | < .001 |
| Segment4 - Segment5 | rock | 0.37 | 0.136 | 48658.408 | 2.718 | 0.051 |

# Smaller dataset pairwise comparisons

*Note:* All p-values are corrected for multiple tests using Tukey’s method.

## Pairwise comparisons of word rates across genres within segment

Table 9. Pairwise comparisons of Cognitive Tension word rates within segments between genres (smaller dataset)

| contrast | Segment | estimate | SE | df | t.ratio | p.value |
| --- | --- | --- | --- | --- | --- | --- |
| country - pop | 1 | -0.848 | 0.167 | 8337.811 | -5.09 | < .001 |
| country - rap | 1 | 0.427 | 0.167 | 8337.811 | 2.557 | 0.052 |
| country - rock | 1 | -0.237 | 0.184 | 8337.811 | -1.29 | 0.569 |
| pop - rap | 1 | 1.275 | 0.168 | 8337.811 | 7.607 | < .001 |
| pop - rock | 1 | 0.611 | 0.184 | 8337.811 | 3.315 | 0.005 |
| rap - rock | 1 | -0.664 | 0.185 | 8337.811 | -3.596 | 0.002 |
| country - pop | 2 | -0.263 | 0.167 | 8337.811 | -1.58 | 0.39 |
| country - rap | 2 | 0.715 | 0.167 | 8337.811 | 4.281 | < .001 |
| country - rock | 2 | 0.08 | 0.184 | 8337.811 | 0.434 | 0.973 |
| pop - rap | 2 | 0.978 | 0.168 | 8337.811 | 5.837 | < .001 |
| pop - rock | 2 | 0.343 | 0.184 | 8337.811 | 1.861 | 0.245 |
| rap - rock | 2 | -0.635 | 0.185 | 8337.811 | -3.441 | 0.003 |
| country - pop | 3 | -0.524 | 0.167 | 8337.811 | -3.148 | 0.009 |
| country - rap | 3 | 0.681 | 0.167 | 8337.811 | 4.078 | < .001 |
| country - rock | 3 | -0.209 | 0.184 | 8337.811 | -1.135 | 0.668 |
| pop - rap | 3 | 1.205 | 0.168 | 8337.811 | 7.193 | < .001 |
| pop - rock | 3 | 0.316 | 0.184 | 8337.811 | 1.714 | 0.316 |
| rap - rock | 3 | -0.89 | 0.185 | 8337.811 | -4.818 | < .001 |
| country - pop | 4 | -0.285 | 0.167 | 8337.811 | -1.714 | 0.316 |
| country - rap | 4 | 0.801 | 0.167 | 8337.811 | 4.797 | < .001 |
| country - rock | 4 | 0.285 | 0.184 | 8337.811 | 1.551 | 0.407 |
| pop - rap | 4 | 1.087 | 0.168 | 8337.811 | 6.486 | < .001 |
| pop - rock | 4 | 0.571 | 0.184 | 8337.811 | 3.097 | 0.011 |
| rap - rock | 4 | -0.516 | 0.185 | 8337.811 | -2.796 | 0.027 |
| country - pop | 5 | -0.136 | 0.167 | 8337.811 | -0.818 | 0.846 |
| country - rap | 5 | 0.494 | 0.167 | 8337.811 | 2.959 | 0.016 |
| country - rock | 5 | 0.177 | 0.184 | 8337.811 | 0.964 | 0.77 |
| pop - rap | 5 | 0.63 | 0.168 | 8337.811 | 3.762 | 0.001 |
| pop - rock | 5 | 0.313 | 0.184 | 8337.811 | 1.701 | 0.323 |
| rap - rock | 5 | -0.317 | 0.185 | 8337.811 | -1.718 | 0.315 |

Table 10. Pairwise comparisons of Plot Progression word rates within segments between genres (smaller dataset)

| contrast | Segment | estimate | SE | df | t.ratio | p.value |
| --- | --- | --- | --- | --- | --- | --- |
| country - pop | 1 | -6.833 | 0.552 | 6402.962 | -12.388 | < .001 |
| country - rap | 1 | 0.184 | 0.553 | 6402.962 | 0.333 | 0.987 |
| country - rock | 1 | -1.922 | 0.609 | 6402.962 | -3.158 | 0.009 |
| pop - rap | 1 | 7.017 | 0.555 | 6402.962 | 12.643 | < .001 |
| pop - rock | 1 | 4.911 | 0.61 | 6402.962 | 8.049 | < .001 |
| rap - rock | 1 | -2.106 | 0.612 | 6402.962 | -3.443 | 0.003 |
| country - pop | 2 | -4.947 | 0.552 | 6402.962 | -8.969 | < .001 |
| country - rap | 2 | 2.328 | 0.553 | 6402.962 | 4.209 | < .001 |
| country - rock | 2 | -0.432 | 0.609 | 6402.962 | -0.71 | 0.893 |
| pop - rap | 2 | 7.275 | 0.555 | 6402.962 | 13.108 | < .001 |
| pop - rock | 2 | 4.515 | 0.61 | 6402.962 | 7.4 | < .001 |
| rap - rock | 2 | -2.76 | 0.612 | 6402.962 | -4.513 | < .001 |
| country - pop | 3 | -4.717 | 0.552 | 6402.962 | -8.552 | < .001 |
| country - rap | 3 | 2.717 | 0.553 | 6402.962 | 4.911 | < .001 |
| country - rock | 3 | -0.369 | 0.609 | 6402.962 | -0.606 | 0.93 |
| pop - rap | 3 | 7.434 | 0.555 | 6402.962 | 13.395 | < .001 |
| pop - rock | 3 | 4.348 | 0.61 | 6402.962 | 7.127 | < .001 |
| rap - rock | 3 | -3.086 | 0.612 | 6402.962 | -5.045 | < .001 |
| country - pop | 4 | -4.487 | 0.552 | 6402.962 | -8.134 | < .001 |
| country - rap | 4 | 3.77 | 0.553 | 6402.962 | 6.815 | < .001 |
| country - rock | 4 | 0.002 | 0.609 | 6402.962 | 0.003 | 1 |
| pop - rap | 4 | 8.257 | 0.555 | 6402.962 | 14.877 | < .001 |
| pop - rock | 4 | 4.488 | 0.61 | 6402.962 | 7.356 | < .001 |
| rap - rock | 4 | -3.769 | 0.612 | 6402.962 | -6.161 | < .001 |
| country - pop | 5 | -4.421 | 0.552 | 6402.962 | -8.016 | < .001 |
| country - rap | 5 | 2.119 | 0.553 | 6402.962 | 3.831 | 0.001 |
| country - rock | 5 | 1.196 | 0.609 | 6402.962 | 1.966 | 0.201 |
| pop - rap | 5 | 6.541 | 0.555 | 6402.962 | 11.785 | < .001 |
| pop - rock | 5 | 5.618 | 0.61 | 6402.962 | 9.207 | < .001 |
| rap - rock | 5 | -0.923 | 0.612 | 6402.962 | -1.509 | 0.432 |

Table 11. Pairwise comparisons of Staging word rates within segments between genres (smaller dataset)

| contrast | Segment | estimate | SE | df | t.ratio | p.value |
| --- | --- | --- | --- | --- | --- | --- |
| country - pop | 1 | 4.137 | 0.328 | 8118.273 | 12.62 | < .001 |
| country - rap | 1 | 4.603 | 0.329 | 8118.273 | 13.998 | < .001 |
| country - rock | 1 | 1.97 | 0.362 | 8118.273 | 5.448 | < .001 |
| pop - rap | 1 | 0.466 | 0.33 | 8118.273 | 1.412 | 0.492 |
| pop - rock | 1 | -2.167 | 0.363 | 8118.273 | -5.975 | < .001 |
| rap - rock | 1 | -2.633 | 0.364 | 8118.273 | -7.242 | < .001 |
| country - pop | 2 | 2.997 | 0.328 | 8118.273 | 9.141 | < .001 |
| country - rap | 2 | 2.457 | 0.329 | 8118.273 | 7.473 | < .001 |
| country - rock | 2 | 1.222 | 0.362 | 8118.273 | 3.38 | 0.004 |
| pop - rap | 2 | -0.539 | 0.33 | 8118.273 | -1.635 | 0.359 |
| pop - rock | 2 | -1.774 | 0.363 | 8118.273 | -4.893 | < .001 |
| rap - rock | 2 | -1.235 | 0.364 | 8118.273 | -3.397 | 0.004 |
| country - pop | 3 | 2.991 | 0.328 | 8118.273 | 9.123 | < .001 |
| country - rap | 3 | 2.719 | 0.329 | 8118.273 | 8.27 | < .001 |
| country - rock | 3 | 1.703 | 0.362 | 8118.273 | 4.708 | < .001 |
| pop - rap | 3 | -0.272 | 0.33 | 8118.273 | -0.823 | 0.844 |
| pop - rock | 3 | -1.288 | 0.363 | 8118.273 | -3.551 | 0.002 |
| rap - rock | 3 | -1.016 | 0.364 | 8118.273 | -2.796 | 0.027 |
| country - pop | 4 | 3.275 | 0.328 | 8118.273 | 9.989 | < .001 |
| country - rap | 4 | 2.161 | 0.329 | 8118.273 | 6.572 | < .001 |
| country - rock | 4 | 1.305 | 0.362 | 8118.273 | 3.609 | 0.002 |
| pop - rap | 4 | -1.114 | 0.33 | 8118.273 | -3.377 | 0.004 |
| pop - rock | 4 | -1.97 | 0.363 | 8118.273 | -5.431 | < .001 |
| rap - rock | 4 | -0.856 | 0.364 | 8118.273 | -2.354 | 0.086 |
| country - pop | 5 | 3.138 | 0.328 | 8118.273 | 9.573 | < .001 |
| country - rap | 5 | 2.314 | 0.329 | 8118.273 | 7.037 | < .001 |
| country - rock | 5 | 1.964 | 0.362 | 8118.273 | 5.429 | < .001 |
| pop - rap | 5 | -0.824 | 0.33 | 8118.273 | -2.499 | 0.06 |
| pop - rock | 5 | -1.174 | 0.363 | 8118.273 | -3.239 | 0.007 |
| rap - rock | 5 | -0.35 | 0.364 | 8118.273 | -0.963 | 0.77 |

## Pairwise comparisons of word rates across segments within genre

Table 12. Pairwise comparisons of Cognitive Tension word rates within genres between segments (smaller dataset)

| contrast | genre | estimate | SE | df | t.ratio | p.value |
| --- | --- | --- | --- | --- | --- | --- |
| Segment1 - Segment2 | country | -0.296 | 0.127 | 11276 | -2.334 | 0.134 |
| Segment1 - Segment3 | country | -0.391 | 0.127 | 11276 | -3.081 | 0.018 |
| Segment1 - Segment4 | country | -0.299 | 0.127 | 11276 | -2.353 | 0.129 |
| Segment1 - Segment5 | country | -0.113 | 0.127 | 11276 | -0.889 | 0.901 |
| Segment2 - Segment3 | country | -0.095 | 0.127 | 11276 | -0.747 | 0.945 |
| Segment2 - Segment4 | country | -0.002 | 0.127 | 11276 | -0.019 | 1 |
| Segment2 - Segment5 | country | 0.183 | 0.127 | 11276 | 1.445 | 0.599 |
| Segment3 - Segment4 | country | 0.092 | 0.127 | 11276 | 0.728 | 0.95 |
| Segment3 - Segment5 | country | 0.278 | 0.127 | 11276 | 2.192 | 0.183 |
| Segment4 - Segment5 | country | 0.186 | 0.127 | 11276 | 1.464 | 0.586 |
| Segment1 - Segment2 | pop | 0.288 | 0.128 | 11276 | 2.258 | 0.159 |
| Segment1 - Segment3 | pop | -0.068 | 0.128 | 11276 | -0.529 | 0.984 |
| Segment1 - Segment4 | pop | 0.264 | 0.128 | 11276 | 2.064 | 0.236 |
| Segment1 - Segment5 | pop | 0.599 | 0.128 | 11276 | 4.687 | < .001 |
| Segment2 - Segment3 | pop | -0.356 | 0.128 | 11276 | -2.786 | 0.043 |
| Segment2 - Segment4 | pop | -0.025 | 0.128 | 11276 | -0.194 | 1 |
| Segment2 - Segment5 | pop | 0.31 | 0.128 | 11276 | 2.429 | 0.108 |
| Segment3 - Segment4 | pop | 0.331 | 0.128 | 11276 | 2.592 | 0.072 |
| Segment3 - Segment5 | pop | 0.666 | 0.128 | 11276 | 5.215 | < .001 |
| Segment4 - Segment5 | pop | 0.335 | 0.128 | 11276 | 2.623 | 0.066 |
| Segment1 - Segment2 | rap | -0.008 | 0.128 | 11276 | -0.064 | 1 |
| Segment1 - Segment3 | rap | -0.137 | 0.128 | 11276 | -1.065 | 0.824 |
| Segment1 - Segment4 | rap | 0.076 | 0.128 | 11276 | 0.589 | 0.977 |
| Segment1 - Segment5 | rap | -0.046 | 0.128 | 11276 | -0.355 | 0.997 |
| Segment2 - Segment3 | rap | -0.129 | 0.128 | 11276 | -1.002 | 0.855 |
| Segment2 - Segment4 | rap | 0.084 | 0.128 | 11276 | 0.652 | 0.966 |
| Segment2 - Segment5 | rap | -0.037 | 0.128 | 11276 | -0.291 | 0.998 |
| Segment3 - Segment4 | rap | 0.213 | 0.128 | 11276 | 1.654 | 0.463 |
| Segment3 - Segment5 | rap | 0.091 | 0.128 | 11276 | 0.71 | 0.954 |
| Segment4 - Segment5 | rap | -0.121 | 0.128 | 11276 | -0.944 | 0.88 |
| Segment1 - Segment2 | rock | 0.02 | 0.153 | 11276 | 0.134 | 1 |
| Segment1 - Segment3 | rock | -0.363 | 0.153 | 11276 | -2.372 | 0.123 |
| Segment1 - Segment4 | rock | 0.223 | 0.153 | 11276 | 1.462 | 0.587 |
| Segment1 - Segment5 | rock | 0.301 | 0.153 | 11276 | 1.971 | 0.28 |
| Segment2 - Segment3 | rock | -0.383 | 0.153 | 11276 | -2.506 | 0.089 |
| Segment2 - Segment4 | rock | 0.203 | 0.153 | 11276 | 1.328 | 0.674 |
| Segment2 - Segment5 | rock | 0.281 | 0.153 | 11276 | 1.837 | 0.352 |
| Segment3 - Segment4 | rock | 0.586 | 0.153 | 11276 | 3.834 | 0.001 |
| Segment3 - Segment5 | rock | 0.664 | 0.153 | 11276 | 4.343 | < .001 |
| Segment4 - Segment5 | rock | 0.078 | 0.153 | 11276 | 0.509 | 0.986 |

Table 13. Pairwise comparisons of Plot Progression word rates within genres between segments (smaller dataset)

| contrast | genre | estimate | SE | df | t.ratio | p.value |
| --- | --- | --- | --- | --- | --- | --- |
| Segment1 - Segment2 | country | -2.546 | 0.37 | 11276 | -6.889 | < .001 |
| Segment1 - Segment3 | country | -2.889 | 0.37 | 11276 | -7.816 | < .001 |
| Segment1 - Segment4 | country | -3.368 | 0.37 | 11276 | -9.113 | < .001 |
| Segment1 - Segment5 | country | -2.945 | 0.37 | 11276 | -7.967 | < .001 |
| Segment2 - Segment3 | country | -0.342 | 0.37 | 11276 | -0.927 | 0.887 |
| Segment2 - Segment4 | country | -0.822 | 0.37 | 11276 | -2.224 | 0.171 |
| Segment2 - Segment5 | country | -0.398 | 0.37 | 11276 | -1.078 | 0.818 |
| Segment3 - Segment4 | country | -0.48 | 0.37 | 11276 | -1.297 | 0.693 |
| Segment3 - Segment5 | country | -0.056 | 0.37 | 11276 | -0.151 | 1 |
| Segment4 - Segment5 | country | 0.424 | 0.37 | 11276 | 1.146 | 0.782 |
| Segment1 - Segment2 | pop | -0.66 | 0.372 | 11276 | -1.775 | 0.388 |
| Segment1 - Segment3 | pop | -0.773 | 0.372 | 11276 | -2.078 | 0.23 |
| Segment1 - Segment4 | pop | -1.022 | 0.372 | 11276 | -2.748 | 0.047 |
| Segment1 - Segment5 | pop | -0.534 | 0.372 | 11276 | -1.434 | 0.605 |
| Segment2 - Segment3 | pop | -0.113 | 0.372 | 11276 | -0.303 | 0.998 |
| Segment2 - Segment4 | pop | -0.362 | 0.372 | 11276 | -0.973 | 0.868 |
| Segment2 - Segment5 | pop | 0.127 | 0.372 | 11276 | 0.341 | 0.997 |
| Segment3 - Segment4 | pop | -0.249 | 0.372 | 11276 | -0.67 | 0.963 |
| Segment3 - Segment5 | pop | 0.24 | 0.372 | 11276 | 0.644 | 0.968 |
| Segment4 - Segment5 | pop | 0.489 | 0.372 | 11276 | 1.314 | 0.683 |
| Segment1 - Segment2 | rap | -0.402 | 0.374 | 11276 | -1.076 | 0.819 |
| Segment1 - Segment3 | rap | -0.356 | 0.374 | 11276 | -0.952 | 0.876 |
| Segment1 - Segment4 | rap | 0.217 | 0.374 | 11276 | 0.581 | 0.978 |
| Segment1 - Segment5 | rap | -1.01 | 0.374 | 11276 | -2.699 | 0.054 |
| Segment2 - Segment3 | rap | 0.046 | 0.374 | 11276 | 0.124 | 1 |
| Segment2 - Segment4 | rap | 0.62 | 0.374 | 11276 | 1.656 | 0.461 |
| Segment2 - Segment5 | rap | -0.607 | 0.374 | 11276 | -1.623 | 0.482 |
| Segment3 - Segment4 | rap | 0.573 | 0.374 | 11276 | 1.532 | 0.541 |
| Segment3 - Segment5 | rap | -0.654 | 0.374 | 11276 | -1.747 | 0.405 |
| Segment4 - Segment5 | rap | -1.227 | 0.374 | 11276 | -3.279 | 0.009 |
| Segment1 - Segment2 | rock | -1.057 | 0.445 | 11276 | -2.374 | 0.123 |
| Segment1 - Segment3 | rock | -1.336 | 0.445 | 11276 | -3.001 | 0.023 |
| Segment1 - Segment4 | rock | -1.445 | 0.445 | 11276 | -3.247 | 0.01 |
| Segment1 - Segment5 | rock | 0.173 | 0.445 | 11276 | 0.389 | 0.995 |
| Segment2 - Segment3 | rock | -0.279 | 0.445 | 11276 | -0.627 | 0.971 |
| Segment2 - Segment4 | rock | -0.389 | 0.445 | 11276 | -0.873 | 0.907 |
| Segment2 - Segment5 | rock | 1.23 | 0.445 | 11276 | 2.763 | 0.045 |
| Segment3 - Segment4 | rock | -0.109 | 0.445 | 11276 | -0.246 | 0.999 |
| Segment3 - Segment5 | rock | 1.509 | 0.445 | 11276 | 3.39 | 0.006 |
| Segment4 - Segment5 | rock | 1.618 | 0.445 | 11276 | 3.636 | 0.003 |

Table 14. Pairwise comparisons of Staging word rates within genres between segments (smaller dataset)

| contrast | genre | estimate | SE | df | t.ratio | p.value |
| --- | --- | --- | --- | --- | --- | --- |
| Segment1 - Segment2 | country | 1.61 | 0.247 | 11276 | 6.521 | < .001 |
| Segment1 - Segment3 | country | 1.589 | 0.247 | 11276 | 6.437 | < .001 |
| Segment1 - Segment4 | country | 1.742 | 0.247 | 11276 | 7.054 | < .001 |
| Segment1 - Segment5 | country | 2.475 | 0.247 | 11276 | 10.025 | < .001 |
| Segment2 - Segment3 | country | -0.021 | 0.247 | 11276 | -0.084 | 1 |
| Segment2 - Segment4 | country | 0.132 | 0.247 | 11276 | 0.533 | 0.984 |
| Segment2 - Segment5 | country | 0.865 | 0.247 | 11276 | 3.505 | 0.004 |
| Segment3 - Segment4 | country | 0.152 | 0.247 | 11276 | 0.617 | 0.972 |
| Segment3 - Segment5 | country | 0.886 | 0.247 | 11276 | 3.589 | 0.003 |
| Segment4 - Segment5 | country | 0.734 | 0.247 | 11276 | 2.972 | 0.025 |
| Segment1 - Segment2 | pop | 0.47 | 0.249 | 11276 | 1.889 | 0.323 |
| Segment1 - Segment3 | pop | 0.443 | 0.249 | 11276 | 1.782 | 0.384 |
| Segment1 - Segment4 | pop | 0.879 | 0.249 | 11276 | 3.538 | 0.004 |
| Segment1 - Segment5 | pop | 1.476 | 0.249 | 11276 | 5.94 | < .001 |
| Segment2 - Segment3 | pop | -0.027 | 0.249 | 11276 | -0.107 | 1 |
| Segment2 - Segment4 | pop | 0.41 | 0.249 | 11276 | 1.649 | 0.466 |
| Segment2 - Segment5 | pop | 1.007 | 0.249 | 11276 | 4.051 | < .001 |
| Segment3 - Segment4 | pop | 0.436 | 0.249 | 11276 | 1.756 | 0.4 |
| Segment3 - Segment5 | pop | 1.033 | 0.249 | 11276 | 4.158 | < .001 |
| Segment4 - Segment5 | pop | 0.597 | 0.249 | 11276 | 2.403 | 0.115 |
| Segment1 - Segment2 | rap | -0.536 | 0.25 | 11276 | -2.142 | 0.202 |
| Segment1 - Segment3 | rap | -0.294 | 0.25 | 11276 | -1.177 | 0.765 |
| Segment1 - Segment4 | rap | -0.7 | 0.25 | 11276 | -2.801 | 0.041 |
| Segment1 - Segment5 | rap | 0.186 | 0.25 | 11276 | 0.746 | 0.946 |
| Segment2 - Segment3 | rap | 0.241 | 0.25 | 11276 | 0.965 | 0.871 |
| Segment2 - Segment4 | rap | -0.165 | 0.25 | 11276 | -0.659 | 0.965 |
| Segment2 - Segment5 | rap | 0.722 | 0.25 | 11276 | 2.888 | 0.032 |
| Segment3 - Segment4 | rap | -0.406 | 0.25 | 11276 | -1.625 | 0.482 |
| Segment3 - Segment5 | rap | 0.481 | 0.25 | 11276 | 1.922 | 0.305 |
| Segment4 - Segment5 | rap | 0.887 | 0.25 | 11276 | 3.547 | 0.004 |
| Segment1 - Segment2 | rock | 0.862 | 0.297 | 11276 | 2.9 | 0.031 |
| Segment1 - Segment3 | rock | 1.322 | 0.297 | 11276 | 4.446 | < .001 |
| Segment1 - Segment4 | rock | 1.077 | 0.297 | 11276 | 3.621 | 0.003 |
| Segment1 - Segment5 | rock | 2.469 | 0.297 | 11276 | 8.303 | < .001 |
| Segment2 - Segment3 | rock | 0.46 | 0.297 | 11276 | 1.546 | 0.532 |
| Segment2 - Segment4 | rock | 0.214 | 0.297 | 11276 | 0.721 | 0.952 |
| Segment2 - Segment5 | rock | 1.607 | 0.297 | 11276 | 5.403 | < .001 |
| Segment3 - Segment4 | rock | -0.245 | 0.297 | 11276 | -0.825 | 0.923 |
| Segment3 - Segment5 | rock | 1.147 | 0.297 | 11276 | 3.857 | 0.001 |
| Segment4 - Segment5 | rock | 1.392 | 0.297 | 11276 | 4.682 | < .001 |
